# Uncovering the potential anti-cancer compounds from *Strobilanthes cusia* against colorectal cancer through network pharmacology and molecular docking

**DOI:** 10.1038/s41598-026-56319-6

**Published:** 2026-06-15

**Authors:** Yogananthan Dhanapal, Duraisamy Sridhar, Sulekha Khute, Paranthaman Subash

**Affiliations:** 1 School of Pharmacy, Joy University, Tirunelveli Dist, 627116 Tamil Nadu India; 2 Department of Pharmacy, Sri Shanmugha College of Pharmacy, Sankari, Salem, 637304 Tamil Nadu India

**Keywords:** *S.cusia*, Andrographolide, Colorectal cancer, CHEK1, Molecular docking, Apoptosis, Biochemistry, Cancer, Chemical biology, Chemistry, Computational biology and bioinformatics, Drug discovery

## Abstract

Colorectal cancer (CRC) remains a major global health challenge owing to its therapeutic resistance and high recurrence rates, underscoring the need for mechanism-based interventions. *Strobilanthes cusia* (Nees) Kuntze, a medicinal herb traditionally used for inflammatory disorders, contains bioactive diterpenoids and alkaloids with anticancer potential. However, the molecular mechanisms underlying this process in CRC remain unclear. This study evaluated sequential Soxhlet extracts of *S. cusia* leaves using integrated in-vitro and in-silico methods. Among the n-hexane, chloroform, ethanol, and aqueous fractions, the ethanolic extract exhibited the highest cytotoxicity against HCT-116 and SW-620 CRC cells, with IC_50_ values of 54.37 and 77.52 µg/mL, respectively. GC–MS analysis identified 13 phytoconstituents, and andrographolide was isolated for the first time from *S. cusia* and structurally characterized using UV–Visible, FTIR, and NMR spectroscopy. Network pharmacology analysis identified checkpoint kinase 1 (CHEK1) as a central target. Molecular docking revealed favorable binding of andrographolide to CHEK1 (− 6.40 kcal/mol), comparable to that of the reference compound (− 6.30 kcal/mol), and molecular dynamics simulations confirmed complex stability. In vitro validation demonstrated dose-dependent apoptosis and proliferation inhibition in both cell lines, with significantly lower IC_50_ values at 48 h (isolated: 10.52 ± 1.91µM; commercial: 8.96 ± 9.35µM) than at 24 h. These findings identify CHEK1 as a mechanistically relevant target and support further translational investigation of *S. cusia-derived* andrographolide in CRC therapy.

**Supplementary Information:**

The online version contains supplementary material available at https://doi.org/10.1038/s41598-026-56319-6.

## Introduction

Colorectal cancer (CRC) is the third most commonly diagnosed cancer and the second leading cause of cancer-related mortality worldwide^1^. Despite significant advancements in chemotherapy, radiotherapy, and surgical interventions, the prognosis of CRC patients remains poor because of recurrence, metastasis, and multidrug resistance. More than 90% of cancer-related deaths are associated with metastatic progression and therapeutic failure caused by enhanced drug efflux, DNA repair mechanisms, and evasion of apoptosis^2, 3, 4^. These limitations highlight the urgent need for the development of novel therapeutic strategies capable of overcoming chemoresistance and improving treatment efficacy. Among emerging therapeutic targets, CHK1 has gained considerable attention due to its central role in the DNA damage response pathway. CHK1 regulates cell cycle arrest and DNA repair following genomic damage, particularly in p53-deficient tumor cells. Inhibition of CHK1 disrupts DNA damage checkpoints, forcing cancer cells to progress through mitosis with unrepaired DNA, ultimately resulting in mitotic catastrophe and apoptosis^5, 6^. Several studies have demonstrated that CHK1 inhibitors sensitize tumor cells to chemotherapy and radiotherapy^7^^,8^^,9^^,10^, thereby establishing CHK1 as a promising therapeutic target in colorectal cancer management^11^.

Natural products derived from medicinal plants continue to serve as valuable sources of anticancer agents because of their chemical diversity and multitarget therapeutic potential. Strobilanthes cusia (*S.cusia*), commonly known as Chinese indigo or Assam indigo, has been traditionally used for the treatment of fever, infections, inflammation, digestive disorders, dysentery, and parasitic diseases^12^^,13^. Phytochemical investigations of *S. cusia*have revealed the presence of biologically active constituents such as alkaloids, flavonoids, terpenoids, fatty acids, indirubin, tryptanthrin, and other secondary metabolites possessing anti-inflammatory, antioxidant, antimicrobial, hepatoprotective, and anticancer properties^14, 15^. Recent studies have reported that compounds derived from *S. cusia*exhibit significant anticancer activity through modulation of apoptosis, inhibition of tumor proliferation, and suppression of inflammatory signaling pathways associated with CRC progression^16^^,17^^,18^^,19^ and Previous studies have demonstrated that andrographolide exerts significant anticancer activity against colorectal cancer by inducing apoptosis, inhibiting PI3K/Akt and NF-κB signaling pathways, suppressing tumor cell proliferation, and enhancing chemosensitivity^20^. Similarly, *S. cusia*and its derived preparations, including indigo naturalis, have been reported to possess anti-inflammatory, antioxidant, and anticancer properties associated with gastrointestinal disorders and colorectal cancer-related pathways^20^. Modern drug discovery approaches have shifted from the conventional “one drug–one target” concept toward multitarget therapeutic strategies suitable for complex diseases such as cancer. In this context, network pharmacology has emerged as an important systems-level approach integrating systems biology, bioinformatics, molecular interactions, and polypharmacology to elucidate the multitarget mechanisms of medicinal plants^17, 18, 19, 21^. By linking phytochemicals with disease-associated genes and signaling pathways, network pharmacology enables the identification of key therapeutic targets and molecular mechanisms involved in disease modulation^17, 18^. This approach is particularly relevant for*S. cusia* because of its rich phytochemical diversity and broad pharmacological properties.

A significant finding of the study was the identification and isolation of andrographolide from *S. cusia*, representing the first report of this diterpenoid lactone in this species. Previously recognized as a characteristic compound of *Andrographis paniculata*, andrographolide was identified in *S. cusia*through phytochemical and spectroscopic analyses, suggesting expanded biosynthetic potential^11^. Furthermore, andrographolide exhibited selective cytotoxic activity against colorectal cancer cells and demonstrated strong interactions with CHK1 during molecular docking and molecular dynamics analyses. Mechanistic studies indicated that andrographolide-mediated CHK1 inhibition resulted in disruption of DNA damage checkpoints and induction of apoptosis in colorectal cancer cells^5, 6^. The present study integrates traditional medicinal knowledge with modern network pharmacology, molecular docking, molecular dynamics simulation, and in vitro validation to demonstrate the anticancer potential of*S. cusia*and its bioactive constituent andrographolide against colorectal cancer^10, 11^.

## Results

### *Strobilanthes cusia* fractions, yield, and cytotoxicity assessment

**Fig. 1:**
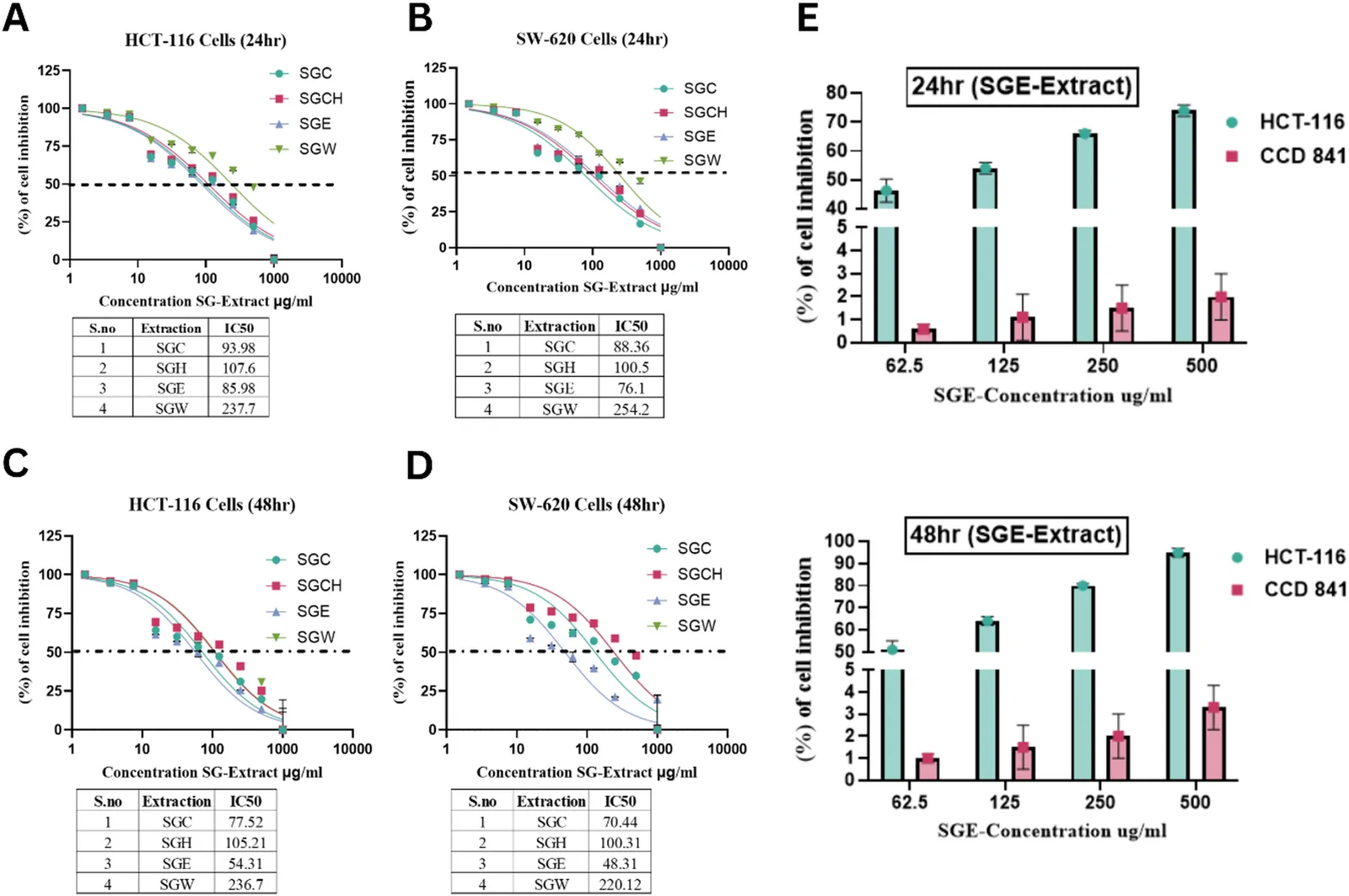
Anti-cancer effect of *S. cusia* extract on colon cancer cells with different mutation types. (**A** and **B**) 24 h treatment of SC different extracts in HCT-116 and SE-620 cells (**C** and **D**), 48 h treatment of SG different extracts in HCT-116 and SE-620 cells. (**E**) Ethanol *S. cusia* extract is more selective for the colorectal carcinoma cell line HT-116 than for the normal lung epithelial cell line CCD 841. To determine the therapeutic index of the ethanol extract, normal colon epithelial cell line CCD 841 and colorectal carcinoma cell line HT-116 were treated with increasing concentrations of the extract for 24 and 48 h. The data shown are the mean ± SEM of three independent experiments with five replicate wells in each experiment.

To identify potential anticancer agents in *S. cusia*, the leaves were sequentially extracted using n-hexane (SCH), chloroform (SCC), 70% ethanol (SCE), and water (SCW). The percentage yields of the extracts are summarized in Table 1, and their anti-cancer potential against CRC cell lines was evaluated using the MTT assay after 24 h of treatment, as shown in Fig. 1A and B. Among the extracts, the ethanol fraction exhibited the strongest cytotoxic activity against HCT-116 and SW-620 cells, with an IC₅₀ value of 54.37 µg/mL, which was significantly lower than those of the chloroform (77.52 µg/mL), n-hexane (105.21 µg/mL), and water (236.07 µg/mL) fractions (Fig. 1C and D). Although the ethanol extract caused only approximately 25% growth inhibition at 500 µg/mL after 24 h, extending the treatment to 48 h enhanced its efficacy to approximately 60%, indicating a time-dependent effect. To evaluate its selectivity, the cytotoxicity of the ethanol extract was compared between normal colorectal epithelial CCD 841 and HCT-116 cells at concentrations ranging from 10 to 500 µg/mL for 24 and 48 h, respectively. The ethanol extract displayed strong selectivity toward cancer cells, producing only 1.02% growth inhibition in CCD 841 cells compared to 55% in HCT-116 cells after 24 h of treatment with 100 µg/mL extract. At 500 µg/mL, the inhibition increased to approximately 3% in CCD 841 cells and 85% in HCT-116 cells. However, prolonged incubation for 48 h slightly reduced selectivity due to mild growth suppression in the normal cells. Collectively, these findings demonstrate that the ethanol extract of *S. cusia* exhibits potent, time-dependent, and selective cytotoxicity toward CRC cells.

**Table 1:** Percentage yield of crude extracts of *Strobilanthes cusia* leaves.

S.no	Solvents	Abbreviation	Percentage yield of SG 100/g of dried leaves (%)
1	70% Ethanol	SCE	8.64 ± 0.02
2	n-Hexane	SCH	3.12 ± 0.15
3	Aqueous/water	SCW	3.53 ± 0.18
4	Chloroform	SCC	4.86 ± 0.10

*S. cusia *ethanol extract *(*SCE), *S. cusia *n-Hexane extract *(*SCH), *S. cusia *Aqueous extract *(*SCW), and *S. cusia *chloroform extract *(*SCC).

### Quantitative phytochemical screening of *S. cusia* ethanolic extract

**Fig. 2:**
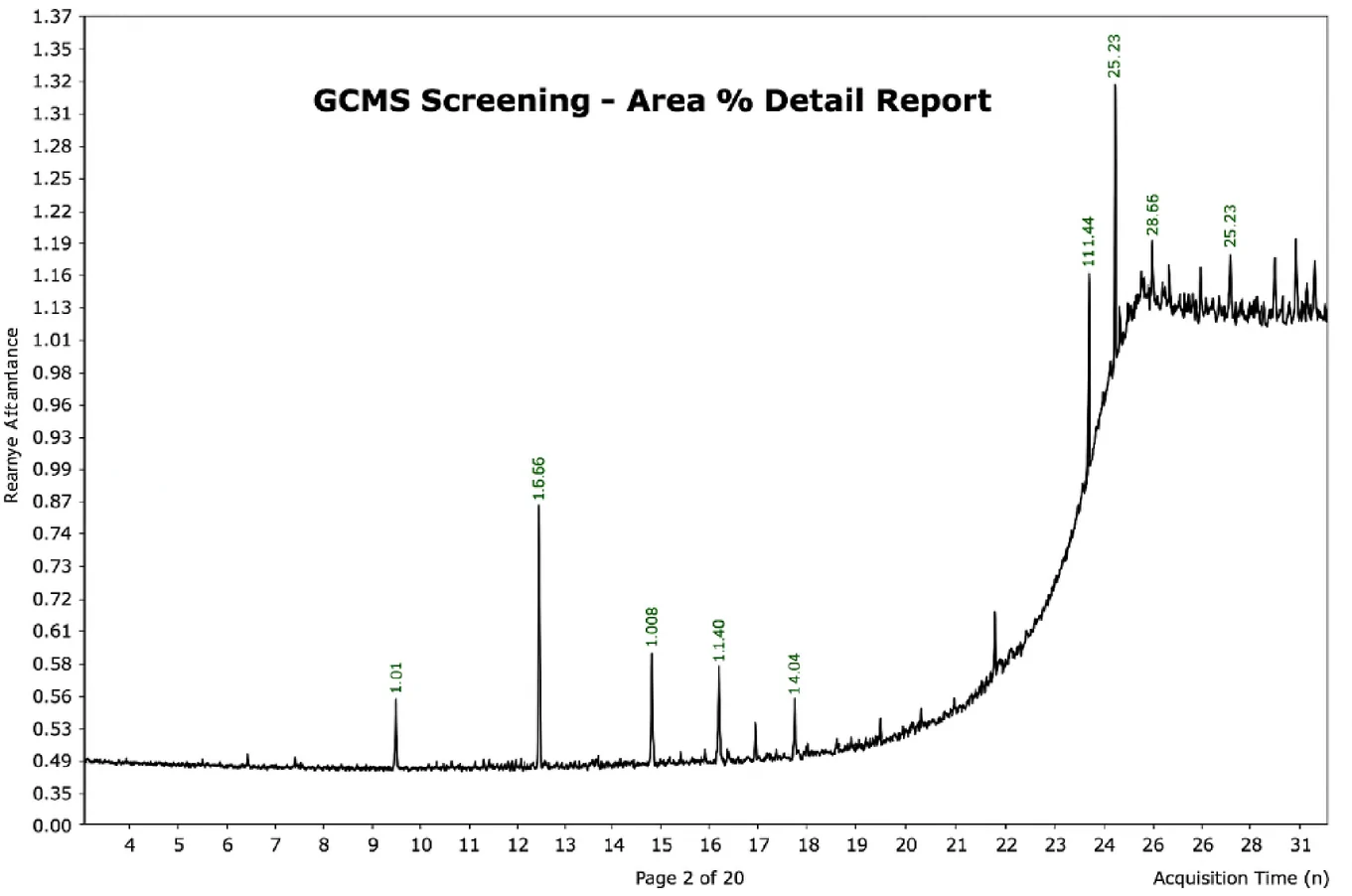
GC-MS chromatogram of the *S. cusia* ethanolic extract.

Gas chromatography–mass spectrometry (GC–MS) is a powerful technique for analyzing complex mixtures and identifying individual compounds in plant extracts. This method separates sample components, detects them based on their mass spectra, and enables the precise identification of phytochemicals. GC–MS is highly reliable and sensitive, making it suitable for the detailed profiling of complex botanical extracts. The 13 phytoconstituents were identified in the ethanolic extract of *S. cusia* leaves using GC–MS analysis, and the resulting chromatogram is presented in Fig. 2. Supplementary Table S1 lists the identified compounds along with their retention times (RT) in the leaf extract. The combination of retention times and mass spectra for each peak provided comprehensive information on the molecular structure and relative abundance of phytoconstituents in the sample.

#### Purification and characterization of anti-cancer agent andrographolide from ethanol extract

**Fig. 3:**
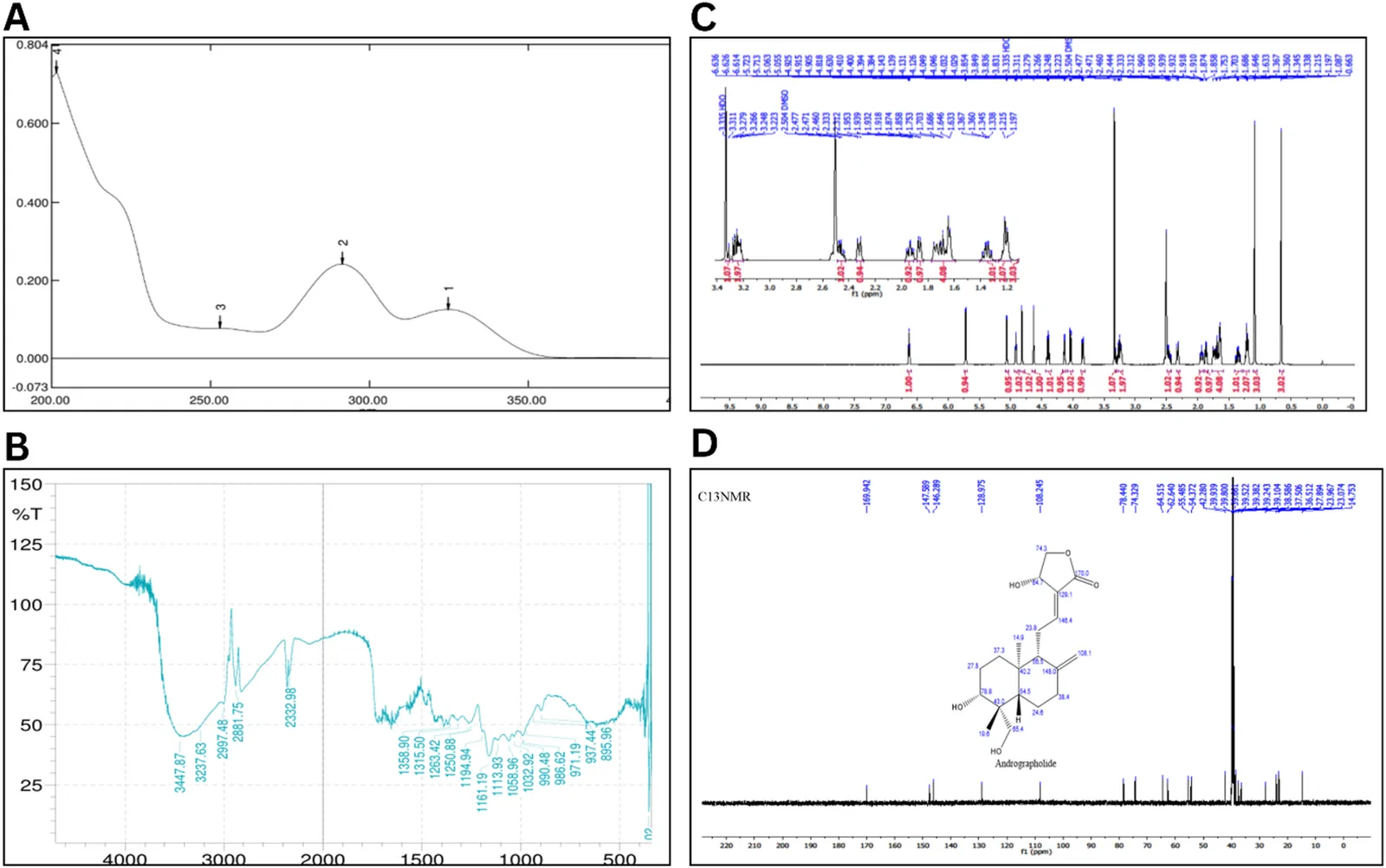
(**A**) UV Spectrum of isolate Andrographolide, (**B**) IR Spectrum, (**C**) 1 H NMR spectra of isolated compound, (**D**) 13 C NMR spectra of isolated compound.

The isolated andrographolide was scanned for absorbance in the range of 190–1000 nm. The maximum absorption was observed at 223 nm (Fig. 3A). These results were comparable to those obtained with standard andrographolide (Sigma-Aldrich, cat. no. 425671) and were consistent with previously reported results. The FTIR results of andrographolide isolated from SC showed bond stretching at (3395) OH-hydroxyl group, (3090 − 2850) alkanes, (1670 − 1650) carbon-carbon double bond, as shown in (Supplementary Table S2). A characteristic stretch of andrographolide corresponding to gamma lactone carbonyl stretching was observed at 1727 cm-1 (Fig. 3B). These data were comparable to those of standard andrographolide and previously reported results (18). 1 H NMR (DMSO-d6, 399.87 MHz) was found δ 6.63 (t, J = 6.6 Hz, 1 H), 5.72 (d, J = 6.1 Hz, 1 H), 5.06 (d, J = 4.9 Hz, 1 H), 4.91 (t, J = 5.9 Hz, 1 H), 4.82 (s, 1 H), 4.63 (s, 1 H), 4.40 (dd, J = 9.9, 6.1 Hz, 1 H), 4.13 (dd, J = 7.5, 2.7 Hz, 1 H), 4.04 (dd, J = 9.9, 1.8 Hz, 1 H), 3.84 (dd, J = 10.9, 2.7 Hz, 1 H), 3.32–3.31 (m, 1 H), 3.28–3.20 (m, 2 H), 2.49–2.42 (m, 1 H), 2.32 (d, J = 12.6 Hz, 1 H), 1.94 (dt, J = 12.8, 6.3 Hz, 1 H), 1.87 (d, J = 9.8 Hz, 1 H), 1.77–1.59 (m, 4 H), 1.35 (qd, J = 13.0, 4.0 Hz, 1 H), 1.21 (d, J = 10.7 Hz, 2 H), 1.09 (s, 3 H), 0.66 (s, 3 H). In addition, the characteristic gamma lactone chiral carbon proton attached to the–OH group resonated at 6.579 ppm as a triplet of doublets (Fig. 3C and Supplementary Table 3). 13 C-NMR (δ 169.94, 147.59, 146.29, 128.98, 108.24, 78.44, 74.33, 64.52, 62.64, 55.48, 54.37, 42.28, 38.59, 37.51, 36.51, 27.89, 23.97, 23.07, 14.75 (Fig. 3D and Supplementary Table 4). The 13 C-NMR data presented for andrographolide isolated from ALw correlated with previous reports.

### Identification of potential targets for colorectal cancer using network pharmacology

#### Identification of biologically active components of *S.cusia*

The phytochemical constituents of *S. cusia* were identified using GC–MS analysis. Thirteen bioactive compounds were detected in the ethanolic extract, representing a diverse range of chemical classes. To assess their drug-likeness and physicochemical suitability for further drug discovery applications, the canonical SMILES of each compound were retrieved and analyzed using the MolSoft database (https://molsoft.com/mprop/). The drug-likeness score of each phytoconstituent was calculated based on molecular descriptors and Lipinski’s rule of five. A comprehensive list of the identified phytoconstituents, along with their corresponding canonical SMILES and drug-likeness scores, is presented in Table 2.

**Table 2:** Identification of *S. cusia phytoconstituent-related* gene using super-PRED.

SMILIES	Gene code
CCCCCCCCCCCCCCCC(OCCCCCCCCCCCC)OCCCCCCCCCCCC	CYP19A1, VEGFA, PSEN2, PSENEN, NCSTN, APH1A PSEN1 APH1B, FGF1, FGF2
CCCCCCCCCCCCCCCCCCCCCCO	TRPM8, CA2, CA1, CA4, ESR1, ESR2, SHBG, CDC25A, NR1I3, NR1H4, UGT2B7, GPBAR1, SHH, AR, SQLE, CDC25B, CNR1, CNR2, POLA1, ACHE, HSD11B1, HMGCR, FAAH, CTSL
CCCCCCCCCCCCCC(CC(CC)CC)CC(CC)CC	SHBG, ACACB
CC(C)(C)C1 = CC(= C(C = C1)O)C(C)(C)C	CA2, PTGS1, HTR2B, HTR2C, GABRA1, GABRB2, GABRG2, SLC6A2, GABRB3, GABRG2, GABRA1, GABBR2, GABBR1, TNNC1, TNNT2, TNNI3, ESR2, HTR6, ESRRG, RORC, ESR1, AR, TRPA1, MC4R, TYR,
CC1(CCCC2(C1 = COC2)C)C	ACHE, SIGMAR1
CCCCCCCCCCCCCCCCCCOC(= O)CCC1 = C(/C/2 = C(/C3 = C(C(= C([N-]3)/C = C\4/C(= C(C([N-]4)/C = C\5/C(= C6C(= O)CC(= C6[N-]5)C1[N-]2)C)CC)C)C(C)O)C)\C)C.[Mg + 2]	D11B1, EPHX2, SIRT2, CA4, SHBG, HDAC6, PGR, MAP2, MAPT, ASAH1, ADRB3, IDO1, EPHX1, CHRM2, CHRM1, HTR3A, HRH4, DRD1, NR3C2, PTK6, MAPK10, DRD5, DRD4, DRD3, TSPO, CDK2.
CCCCCC = CCC=CCCCCCCCCOCCO	GPR119, S1PR3, S1PR1, NPC1L1, MDM2, MET, VCP, MAPK14, PTPN1, CDK1, ICMT, APP, NR1H2, ACACB, PPARA, KDR, PIK3CB, LIMK2, RBP4, PSEN2, PSENEN, NCSTN, APH1A, PSEN1, APH1B, HIF1A,
C1 = CC=C2C(= C1)N = C(S2)SCCO	ACHE, HTR2A, HTR1A, HMOX1, HTR7, MAPK14, MAOA, MPO, PRKCD, PRKCE, PRKCH, PRKCQ, CHRM1, AURKA, ADORA1, PARP1, MAP2K1, SLC6A2, FDFT1, ASAH1, MKNK1, PPP1CA, CDK1,
CCCCCCCC/C = C\CCCCCCCC(= O)O	FABP3, SCD, PTPN1, PTPN2, PTGS1, FFAR1, PTGES, TOP1, PREP, PTPN6, ALOX5, CDC25B, CDC25A, AKR1B10, POLB, PDE4D, HMGCR, PTGER2, LTB4R, HSD11B1, RORC, CYP19A1, NR1H3, NR3C1,
CCOC(= O)CC[C@@H](C)[C@H]1CC[C@@H]2[C@@]1([C@H](C[C@H]3[C@H]2[C@@H](C[C@H]4[C@@]3(CC[C@H](C4)O)C)O)O)C	VDR, PTPN1, HMGCR, UGT2B7, CDC25A, CYP19A1, HSD11B1, CA2, CA1, ACHE, NR1H4, TTL, SIRT2, CCR1, P2RX3, MAPK14, PDE10A, HCRTR2, HCRTR1, F10, PTGS2, NR1I2, HSP90AA1, F2R, PTGS1,
CC1 = C(C2 = C(CCC(O2)(C)CCCC(C)CCCC(C)CCCC(C)C)C(= C1O)C)C	AKT1, PHLPP1, ILK, CNR1, CNR2, PSEN1, ESR1, ESR2, PTGS2, ALOX5, PTPN1, PTGES, KDR, PTGS1, PLA2G7, MCHR1, CCKAR, CTSS, PDE10A, MAPK10, and TSPO.
C[C@@]12CC[C@H]([C@@]([C@H]1CCC(= C)[C@H]2 C/C = C/3\[C@@H](COC3 = O)O)(C)CO)O	PRKCA, TTL, PRKCD, IL6, ADAM17, GLUL, GPBAR1, FABP1, AR, JAK1, PDCD4, EIF2AK3, NR3C2, PRKCE, POLA1, PGR, BRD4, PYGL, CREBBP, CA7, CA12, JAK2, DPP4, SLC5A4, CDK1, SLC5A1, CA2, MAP3K5, EPHX2, IMPDH1, IMPDH2, CDK2, PTGS1, WEE1, ALPL, OPRM1, PDE10A, MAP2K1, NEK1, CHRM1, MAPK14, CHRNA4, RBP4, CDK9, CCNE1, CDK2, CHEK1, MMP9, IRAK4, MKNK2, CDK6, CDK4, CDK5, CDK3, CYP19A1, FLT3, PLK4, SLC18A3, AXL, IARS, SLC5A2, OPRL1, PSEN2, PSENEN, NCSTN, APH1A, PSEN1, APH1B.
CCCCCCCCCCCCCC(= O)O[C@@H]1[C@H]([C@]2([C@@H](C = C(C[C@]3([C@H]2 C = C(C3 = O)C)O)CO)[C@H]4[C@@]1(C4(C)C)OC(= O)C)O)C	PRKCG, PRKCD, PRKCA, PRKCB, PRKCE, PRKCQ, VAV1, PRKCH, TRPV4, PTGS2, TERT, ATP2A1, KCNA3, NOS2, JUN, PGGT1B, FNTA, FNTB, F2RL1, PDE4D, NR3C2, PTPA, IARS, GSTM1, PPP1CC,

#### Identification of genes associated with CRC cancer and phytoconstituents

A total of 25,500 CRC-associated genes were initially retrieved from the GeneCards database using the keyword “colorectal cancer.” To enhance the specificity and biological relevance of the dataset, only genes with GeneCards Inferred Functionality Scores (GIFtS) > 60 were retained for downstream analysis, following previously established recommendations for network pharmacology-based herbal medicine studies. Among the filtered genes, CDK1 (Cyclin-Dependent Kinase 1) and JAK1 (Janus Kinase 1) exhibited the highest relevance scores of 156.86 and 100.63, respectively, indicating their strong association with CRC progression and tumor-related signaling pathways. A comprehensive list of CRC-associated targets is provided in Supplementary Table S5. Phytoconstituent-related targets were predicted using the Super-PRED database based on structural similarity, pharmacophore matching, and validated ligand–target interaction profiles. Following the removal of duplicate entries, a total of 142 unique protein targets associated with the identified phytoconstituents of *S.cusia* were obtained. These predicted targets were primarily involved in critical oncogenic processes, including cell proliferation, apoptosis, DNA damage response, inflammatory signaling, kinase activation, and cell cycle regulation. Notably, several CRC-associated targets, such as CDK1, CDK2, CHEK1, CDC25A, CDC25B, JAK1, MAPK14, PTGS1, PTGS2, and WEE1, were identified among the predicted targets, suggesting the multitarget therapeutic potential of *S. cusia*phytoconstituents against CRC. The network pharmacology workflow, including target screening, compound–target interaction analysis, protein–protein interaction network construction, and enrichment analysis, was designed, conducted, and interpreted in accordance with previously established international recommendations for herbal medicine-based network pharmacology research^22^ to ensure methodological transparency, reproducibility, and standardized data interpretation. The predicted compound–target interactions are presented in Table 2.

#### Identification of common genes/proteins associated with both compound and disease

**Fig. 4:**
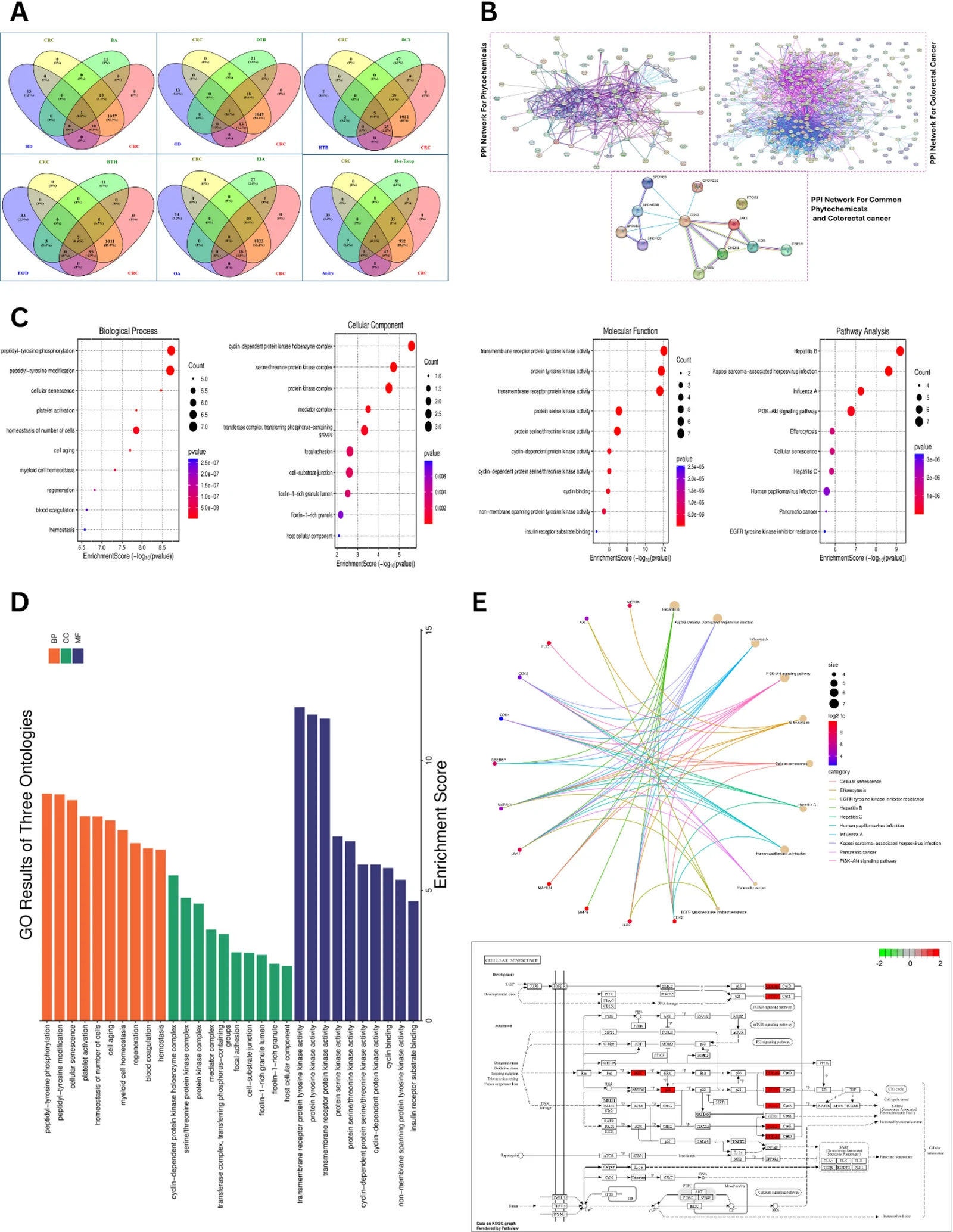
Network pharmacology-based screening of active targets and pathways of *S. cusia* extract against CRC (**A**) Venn diagram illustrating the intersection of disease-related targets and predicted targets of the extract (**B**) PPI network showing interactions among phytoconstituents, CRC, and common targets (**C**) GO and KEGG enrichment analysis of target genes related to *S. cusia* bioactive compounds against CRC (**D**) biological processes, cellular components, and molecular function (D) Pathway analysis.

Venny v2.1 (https://bioinfogp.cnb.csic.es/tools/venny/) was used to identify overlapping gene CRC and phytoconstituents derived from *S. cusia*. Disease-associated genes were retrieved from the GeneCards database, and compound-related targets were predicted using Super-PRED. Comparative analysis of these datasets revealed five shared gene targets, CDK2, CHEK1, CDC25A, CDC25B, and ESR1, which play essential roles in CRC development and progression. Specifically, CDK2 and CDC25A/B are critical regulators of the cell cycle, governing the G1/S and G2/M transitions, and their dysregulation promotes uncontrolled cell proliferation in CRC. CHEK1 is a key component of the DNA damage response pathway that ensures genomic stability by arresting cell division in response to replication stress, a process often exploited by cancer cells for survival. ESR1, although traditionally associated with hormone-responsive cancers, has been implicated in CRC via cross-talk with growth factor signalling pathways, such as PI3K/Akt and MAPK, contributing to tumor progression and chemoresistance. These overlapping targets represent crucial molecular nodes that may mediate the anticancer potential of *S. cusia* bioactive compounds by modulating pathways related to cell cycle control, DNA repair, and receptor-mediated transcription. A Venn diagram generated using Venny v2.1 (Fig. 4A) visually illustrates the intersection between disease- and phytoconstituent-associated genes in the network analysis. The identified targets were subsequently used to construct a protein–protein interaction (PPI) network in Cytoscape, enabling the visualization of their functional connectivity and biological significance within the CRC signalling landscape.

#### Construction and analysis of target PPI network

To elucidate the molecular interactions and underlying mechanisms of the identified target genes, a high-confidence PPI network was constructed using STRING v12, with a confidence score threshold of > 0.9. Nodes represent proteins or hub genes, whereas node colors indicate different interaction categories and hub-gene ranking scores. Edge thickness and interaction line intensity correspond to the confidence strength of PPI obtained from STRING analysis, with thicker edges representing stronger and higher-confidence interactions. The resulting PPI network for the phytoconstituents of *S.cusia* contained 121 nodes and 506 edges, while the CRC network comprised 155 nodes and 2641 edges (Fig. 4B). Each edge represents an interaction between proteins, with an average node degree of 2.31 and a local clustering coefficient of 0.339, indicating a well-connected and functionally relevant network. The common PPI network shared between the phytoconstituent targets and CRC-associated genes consisted of 11 nodes and 13 edges, highlighting key overlapping interactions. Among these, CDK1 emerged as the most influential hub, forming strong and high-confidence interactions with CDK2, CHEK1, CDC25A, CDC25B, and ESR1, as indicated by the thicker connecting lines in the STRING visualization. Subsequent functional enrichment analysis identified nine significantly enriched KEGG pathways, of which 15 pathways were found to be highly relevant to CRC, ranked by P-value to reflect the statistical significance of the association between network proteins and biological pathways. Notably, the PI3K–Akt signalling pathway showed the strongest enrichment, involving critical genes such as KDR, CDK2, CSF1R, and JAK1, suggesting its pivotal role in CRC pathogenesis and as a therapeutic target for CRC. Further Gene Ontology (GO) analysis classified these genes into biological processes, cellular components, and molecular functions, revealing their involvement in cell cycle regulation, apoptosis, and signal transduction. Finally, CytoHubba analysis was performed to rank the most significant hub genes, and the top candidates identified through enrichment and network analyses were prioritized for molecular docking to explore potential ligand–target interactions and validate their mechanistic relevance in colorectal cancer.

#### GO enrichment analysis for biological process, cellular component, and molecular functions

GO enrichment analysis of the nine selected genes was carried out using ShinyGO v0.80. The analysis revealed three key biological processes with high statistical significance, which are highlighted as large red dots in Fig. 4C. This analysis emphasizes the functional roles that are most relevant to colorectal cancer. Notably, the enriched biological processes included the regulation of cell death, both positive and negative, as well as apoptotic signaling pathways, reflecting active host cell turnover and immune response mechanisms. Peptide tyrosine phosphorylation has profound biological consequences, as it functions as a molecular switch that controls nearly every aspect of cell behavior, linking extracellular cues to intracellular responses with remarkable specificity. When a receptor or non-receptor tyrosine kinase phosphorylates target proteins, the added phosphate groups create high-affinity docking sites for signalling domains. The cellular response to CDKs involves serine/threonine kinases that regulate orderly cell cycle progression by phosphorylating specific substrates that are bound to cyclins. However, in cancer, their activity is frequently deregulated through cyclin overexpression, CDK gene amplification, or loss of endogenous inhibitors such as p16 and p21, leading to unchecked G1–S and G2–M transitions, genomic instability, sustained proliferation, and resistance to apoptosis, making aberrant CDK signalling a hallmark of tumorigenesis and a major therapeutic target. CDK4/6 inhibitors are already in clinical use to restrain malignant growth in breast cancers. Molecular functions Transmembrane receptor protein tyrosine kinases drive cancer when mutations, amplifications, or overexpression cause constant activation without ligand binding, leading to persistent signalling through pathways such as MAPK, PI3K–AKT, and JAK–STAT, which in turn promotes uncontrolled cell proliferation, survival, angiogenesis, and metastasis, making aberrant RTK activity a central mechanism of tumor development and a critical target for anticancer therapies that regulate and grow signalling in the inflammatory microenvironment. The network of enriched GO terms, including biological processes, cellular components, and molecular functions, is presented in Fig. 4D. Comparison of GO enrichment with KEGG pathway analysis revealed that JAK1, WEE1, CHEK1, and SPDYE16 were common to both analyses (Fig. 4E). These genes were identified as key candidates for further investigation because of their established roles in colorectal cancer and potential interactions with the selected phytoconstituents.

#### Cytoscape

**Fig. 5:**
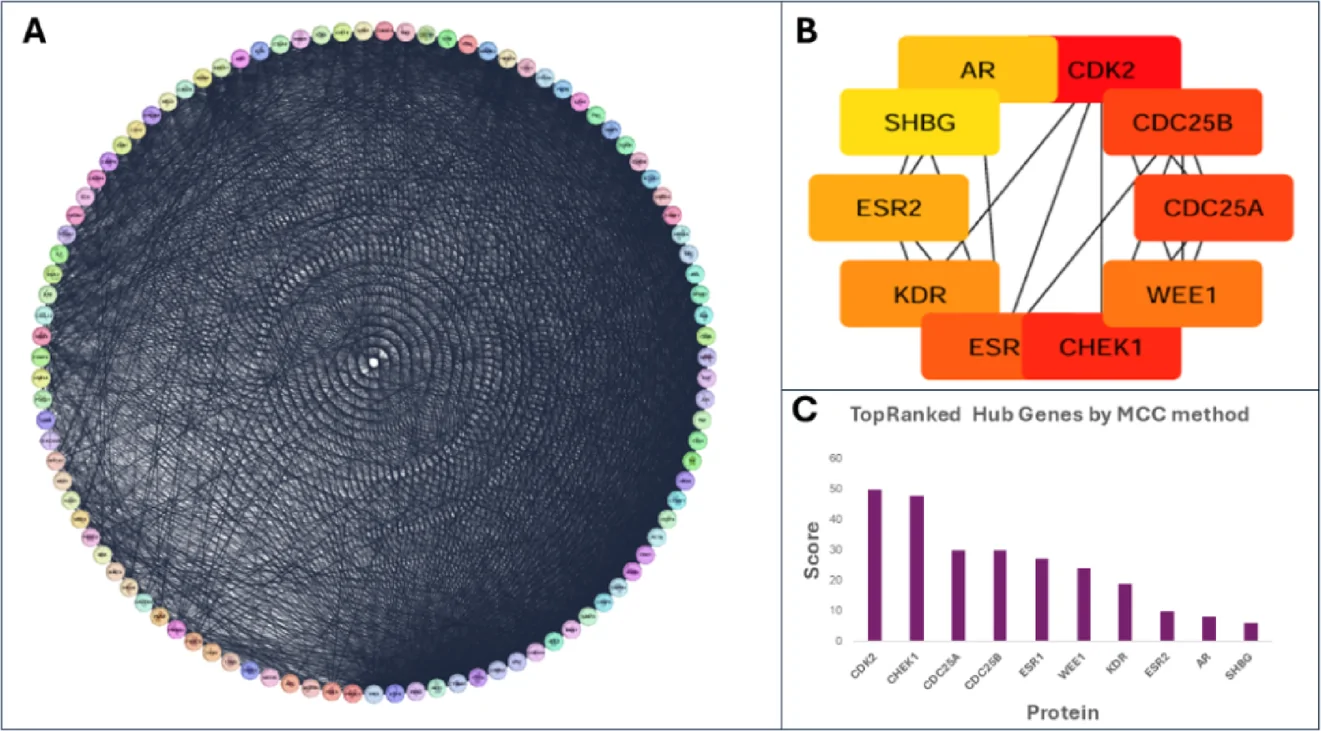
Identification and ranking of hub genes related to *S. cusia* extract in the treatment of CRC (**A**) Core target network constructed based on topological analysis of key proteins (**B**) PPI subnetwork showing the top 10 hub genes based on network centrality where node colors represent hub-gene ranking scores based on MCC analysis and edge thickness indicates interaction confidence and network connectivity strength (**C**) Bar chart representing the top 10 ranked proteins using the MCC algorithm.

PPI network analysis was conducted to elucidate the molecular mechanisms underlying CRC and identify key regulatory targets influenced by *S.cusia* phytoconstituents. Using the CytoHubba plugin in Cytoscape, the Maximum Clique Centrality (MCC) algorithm ranked the most significant hub genes based on their topological importance in the network. The PPI network, comprising 20 nodes and 165 edges, revealed strong interconnectivity among CRC-associated and phytoconstituent-related genes, with the color intensity from red to yellow in Fig. 5 indicating the ranking hierarchy from higher to lower centrality. The top five hub genes identified were CDK2, CHEK1, CDC25A, CDC25B, and ESR1, all of which play crucial roles in cell cycle regulation and DNA damage response. Other important nodes included WEE1, KDR, ESR2, AR, and SHBG, indicating their involvement in angiogenic and hormone-regulated signaling pathways. Collectively, these results highlight CDK2 and CHEK1 as the principal hub genes within the CRC-phytoconstituent network, suggesting that *S. cusia* bioactive compounds may exert anti-cancer effects by modulating cell cycle checkpoint kinases and their associated regulatory pathways.

### Molecular docking analysis

**Fig. 6:**
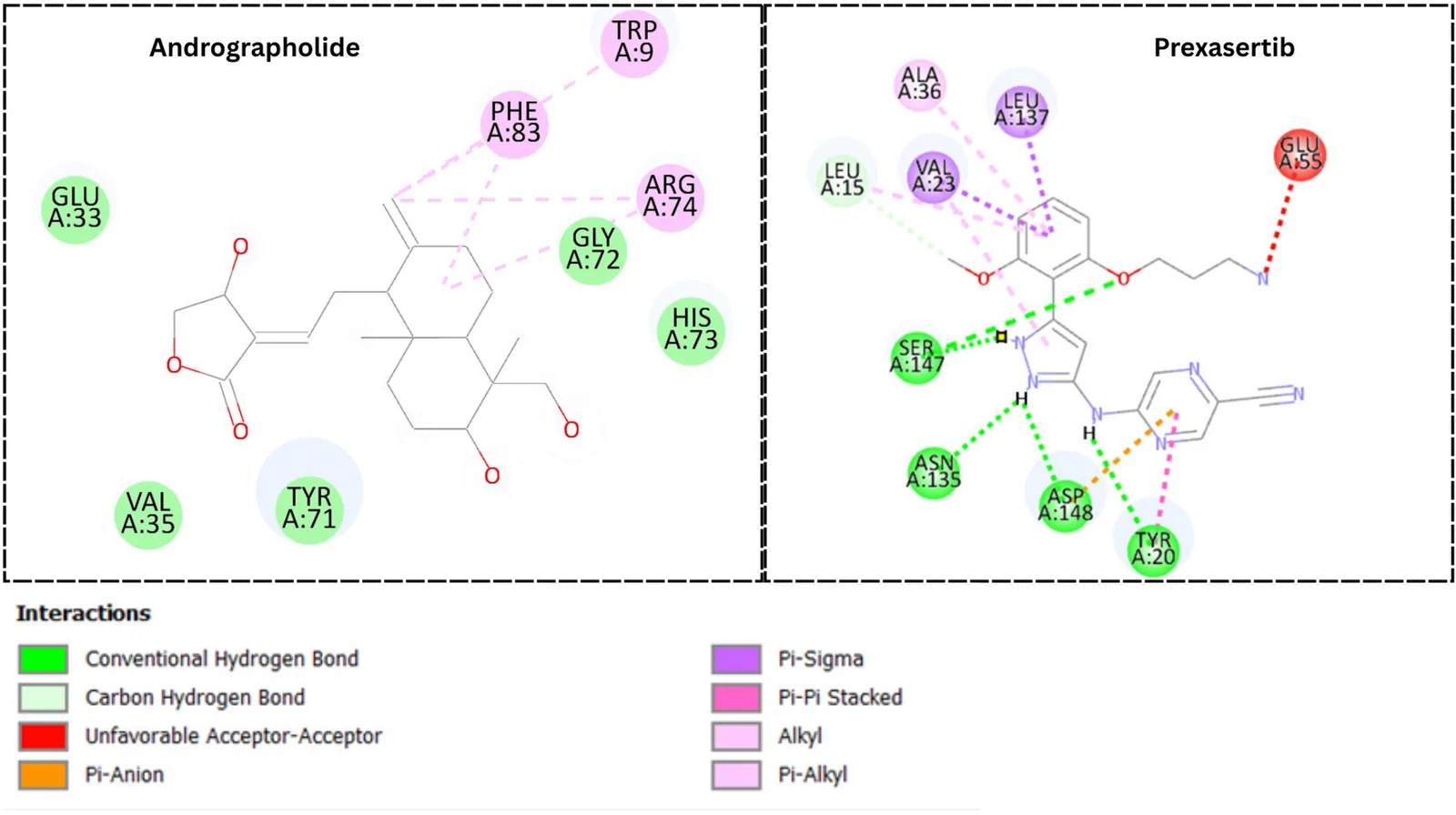
Molecular Docking analysis of the 2D projection of andrographolide and prexasertib interactions with CHEK1 protein.

Molecular docking analysis revealed that among the screened ligands, andrographolide tri-(trimethylsilyl)- exhibited the strongest binding affinity towards the target protein, with a docking score of − 8.50 kcal/mol. The compound formed hydrogen bond interactions with TYR A:31 and ASN A:28 residues and van der Waals interactions with GLU A:33, VAL A:35, TYR A:71, GLY A:72, and HIS A:73, indicating stable accommodation within the active-binding pocket. Ethyl iso-allocholate also demonstrated a high binding affinity of − 6.30 kcal/mol, which was stabilized by a hydrogen bond interaction with ASN A:28 and van der Waals interactions involving THR A:31, GLU A:33, TYR A:71, HIS A:73, and GLY A:72 residues. Similarly, 2,4-Di-tert-butylphenol exhibited a binding affinity of − 6.20 kcal/mol and formed van der Waals interactions with ARG A:74, GLY A:72, and GLU A:33. Dl-α-Tocopherol demonstrated moderate binding affinity with a docking score of − 5.50 kcal/mol and formed a hydrogen bond interaction with GLU A:33, along with van der Waals interactions involving TRP A:9, ARG A:74, GLY A:72, GLU A:7, and LYS A:60 residues. Benzothiazole, 2-(2-hydroxyethylthio)- showed a docking score of − 5.30 kcal/mol and established hydrogen bond interactions with TRP A:9 and ASP A:8, while additional van der Waals contacts were observed with ILE A:39, PHE A:83, ASP A:10, and ARG A:29. Furthermore, 6-Hydroxy-4,4,7a-trimethyltetrahydrobenzofuran-2(4 H)-one exhibited a binding affinity of − 5.70 kcal/mol, which was stabilized by a hydrogen bond interaction with TRP A:9 and van der Waals interactions with ASP A:8 and GLU A:7 residues. Oleic acid displayed a docking score of − 4.90 kcal/mol and formed hydrogen bond interactions with THR A:31 and ASN A:28, accompanied by van der Waals interactions involving VAL A:6, GLU A:7, VAL A:35, and GLU A:33. Behenic alcohol demonstrated a comparatively weaker binding affinity, with a docking score of − 4.50 kcal/mol, and formed a hydrogen bond interaction with GLU A:33, along with van der Waals interactions involving VAL A:6, ASN A:28, THR A:31, VAL A:35, HIS A:73, and GLY A:72 residues. Finally, Ethanol, 2-(9,12-octadecadienyloxy)-, (Z, Z)- exhibited a docking score of − 4.60 kcal/mol without any hydrogen bond interactions; however, it maintained van der Waals interactions with VAL A:6, GLU A:7, ASN A:28, GLU A:33, HIS A:73, and GLY A:72 residues. The standard compound prexasertib exhibited a docking score of − 7.80 kcal/mol and formed multiple hydrogen bond interactions with SER147, ASN135, ASP148, and TYR20 residues, whereas van der Waals interactions were observed with ALA36, VAL23, and LEU137 residues. The docking findings indicate that Andrographolide, tri-(trimethylsilyl)-, and ethyl iso-allocholate exhibit promising inhibitory potential owing to their strong binding affinity and stable interactions with critical amino acid residues within the active site of the target protein. Table 3 presents the docking scores and interaction profiles of the selected ligands, and Fig. 6 and Supplementary Figure S2 illustrates the two-dimensional protein–ligand interaction patterns.

**Table 3:** Comparative analysis of binding strength and binding energy of *S.cusia* Phytoconstituents.

S.no	Ligand	Best Affinity	Hydrogen Bond Interactions	van der Waals Interactions
1	Benzothiazole, 2-(2-hydroxyethylthio)-	–5.30	TRP A:9, ASP A:8	ILE A:39, PHE A:83, ASP A:10, ARG, A:29
2	2,4-Di-tert-butylphenol	–6.20		ARG A:74, GLY A:72, GLU A:33
3	Dl-α-Tocopherol	–5.50	GLU A:33	TRP A:9, ARG A:74, GLY A:72, GLU A:7, LYS A:60
4	Behenic alcohol	–4.50	GLU A:33	VAL A:6, ASN A:28, THR A:31, VAL A:35, HIS A:73, GLY A:72
5	Oleic Acid	–4.90	THR A:31, ASN A:28	VAL A:6, GLU A:7, VAL A:35, GLU A:33.
6	Ethyl iso-allocholate	–6.30	ASN A:28	THR A:31, GLU A:33, TYR A:71, HIS A:73, GLY A:72
7	6-Hydroxy-4,4,7a-trimethyltetrahydrobenzofuran-2(4 H)-one	–5.70	TRP A:9	ASP A:8, GLU A:7
8	Ethanol, 2-(9,12-octadecadienyloxy)-, (Z, Z)-	–4.60	-	VAL A:6, GLU A:7, ASN A:28, GLU A:33, HIS A:73, GLY A:72
9	Andrographolide, tri-(trimethylsilyl)-	–8.50	TYR A:31, ASN A:28	GLU A:33, VAL A:35, TYR A:71, GLY A:72, HIS A:73
10	Prexasertib (Standard)	−7.80	SER147, ASN135, ASP148, and TYR20	ALA36, VAL23, and LEU137

#### In-silico drug-likeness and ADME-tox predictions

ADMET-based pharmacological evaluation of the selected phytoconstituents was performed to determine their drug-likeness and pharmacokinetic suitability as potential lead compounds against the target protein. Key molecular descriptors, including molecular weight, partition coefficient (LogP), molar refractivity, hydrogen bond donors (HBD), hydrogen bond acceptors (HBA), gastrointestinal (GI) absorption, blood–brain barrier (BBB) permeability, and Veber violations, were analyzed to predict membrane permeability, oral bioavailability, and overall pharmacokinetic behavior. The results indicated that compounds with balanced physicochemical properties exhibited improved pharmacodynamic potential and better compliance with Lipinski’s Rule of Five. Among the screened compounds, Andrographolide, tri-(trimethylsilyl)-, Ethyl iso-allocholate, Dl.-alpha.-Tocopherol, and the standard compound Prexasertib demonstrated comparatively favorable pharmacokinetic profiles. Ethyl iso-allocholate and Dl.-alpha.-Tocopherol exhibited high GI absorption, BBB permeability, absence of Veber violations, and good drug-likeness properties, indicating their potential suitability as therapeutic lead molecules. Similarly, Prexasertib showed balanced ADMET characteristics with high GI absorption and acceptable drug-likeness, supporting its effectiveness as a standard inhibitor. Andrographolide, tri-(trimethylsilyl)-, which exhibited the highest docking affinity in molecular docking analysis, demonstrated high GI absorption and strong target-binding potential. The compound possessed three hydrogen bond donors, eight hydrogen bond acceptors, and acceptable lipophilicity, which may contribute to stable protein–ligand interactions. However, the presence of one Veber violation and lack of BBB permeability may influence its pharmacokinetic performance despite its superior docking affinity. Additionally, 2,4-Di-tert-butylphenol and Benzothiazole, 2-(2-hydroxyethylthio)- displayed moderate lipophilicity, favorable hydrogen-bonding characteristics, high GI absorption, and BBB permeability, indicating acceptable pharmacokinetic properties. In contrast, oleic acid and ethanol, 2-(9,12-octadecadienyloxy)-, (Z, Z)- exhibited comparatively low GI absorption and poor drug-likeness despite moderate physicochemical characteristics. Long-chain aliphatic compounds, including hexadecane, 1,1-bis(dodecyloxy)-, octadecane derivatives, and behenic alcohol, demonstrated poor pharmacokinetic suitability owing to excessive hydrophobicity, elevated LogP values, and low GI absorption. Overall, ADMET profiling identified andrographolide, tri-(trimethylsilyl)-, ethyl iso-allocholate, D-l-alpha-Tocopherol, and Prexasertib as the most promising compounds with favorable pharmacokinetic and drug-like properties. These findings support the potential of these compounds for further molecular docking refinement, molecular dynamics simulations, and experimental validation studies. Table 4 summarizes the physicochemical and ADMET properties of selected phytoconstituents.

**Table 4:** ADME prediction of *S. cusia* lead phytoconstituents.

Compound name	Molecular weight	logP	hbd	hba	Molar_refractivity	Veber_violations	Gi absorption	bbb_permeant	Drug_likeness
1. Hexadecane, 1,1-bis(dodecyloxy)-	566.6002	13.541	0	2	180.708	1	Low	No	Poor
2. Behenic alcohol	296.3443	8.4381	0	0	99.071	1	Low	Yes	Poor
3. Octadecane, 3-ethyl-5-(2-ethylbutyl)-	338.3913	9.3202	0	0	112.782	1	Low	Yes	Poor
4. 2,4-Di-tert-butylphenol	206.1671	3.9872	1	1	65.5068	0	High	Yes	Poor
5. Hexadecane, 1,1-bis(dodecyloxy)-	184.1099	1.099	1	3	47.0088	0	High	Yes	Poor
6. Octadecane, 3-ethyl-5-(2-ethylbutyl)-	840.5415	11.33334	1	4	251.7513	1	Low	No	Poor
7. Ethanol, 2-(9,12-octadecadienyloxy)-, (Z, Z)-	254.2246	4.2484	1	2	78.7948	1	Low	Yes	Poor
8. Benzothiazole, 2-(2-hydroxyethylthio)-	211.0126	2.3807	1	4	57.4298	0	High	Yes	Poor
9. Oleic Acid	268.2402	5.7184	1	1	82.4708	1	Low	Yes	Poor
10. Ethyl iso-allocholate	350.2457	3.15	2	4	94.6296	0	High	Yes	Good
11. Octadecane, 3-ethyl-5-(2-ethylbutyl)-	410.3185	6.9641	1	2	126.1018	0	High	No	Poor
12. Dl.-alpha.-Tocopherol	362.2093	2.571	2	5	95.8726	0	High	Yes	Good
13. Andrographolide, tri-(trimethylsilyl)-	616.3975	5.7529	3	8	167.3194	1	High	No	Poor
14. Prexasertib (Standard)	365.39	7.56	3	7	99.18	0	High	no	Good

#### MD simulation analysis

**Fig. 7:**
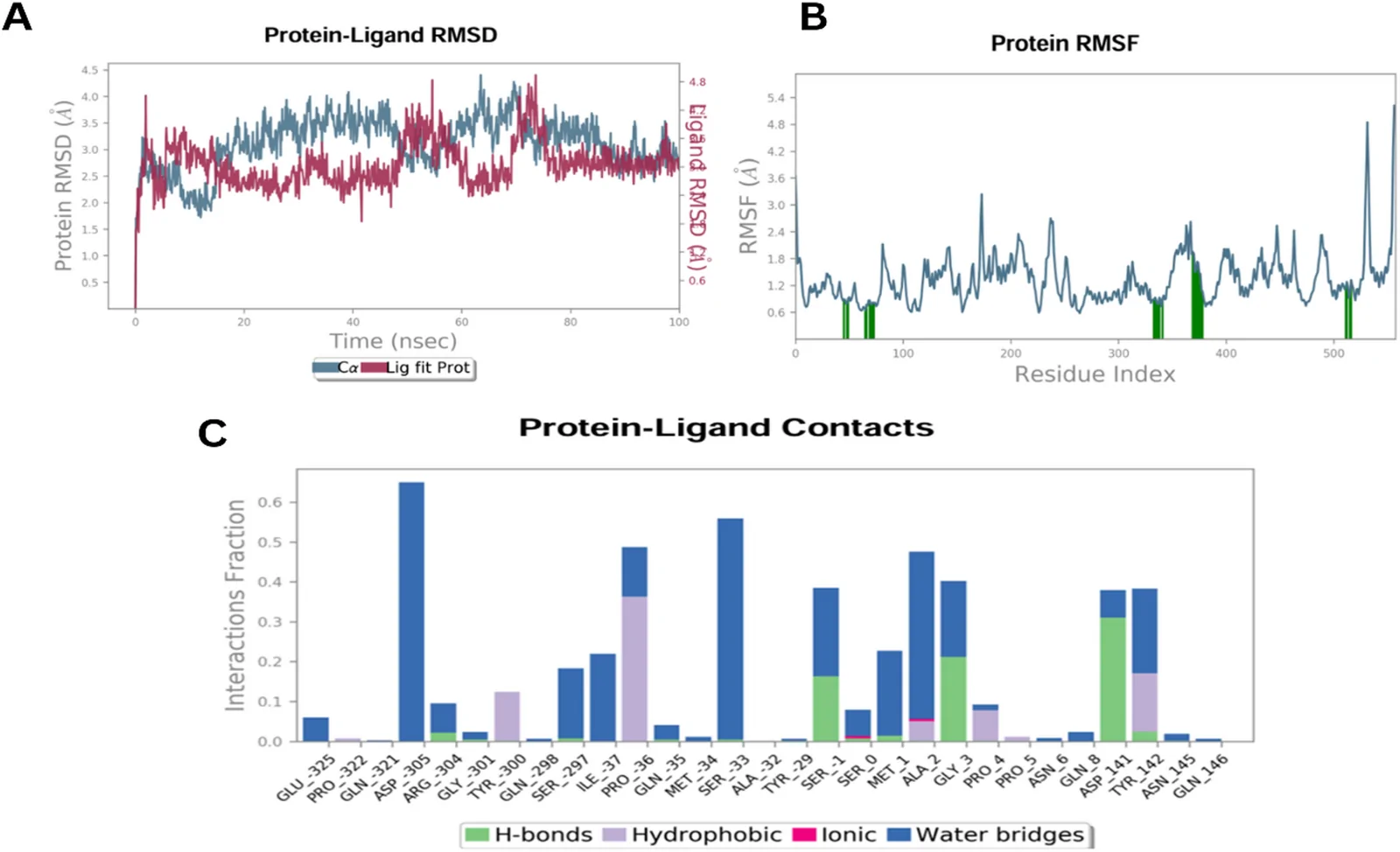
(**A**) RMSD, (**B**) Protein RMSF with Andrographolide, (**C**) Protein Ligand Interaction.

RMSD shows whether the system has reached stability by observing the fluctuation until the value is not significant enough after reaching a specific period. If the RMSD fluctuated significantly until a specific time, the system was considered unstable. The RMSD of the C and the side chain of the protein during the simulation with *S.cusia* ligands is shown in Fig. 7A. The RMSD value was used to compare the docked and reference ligand conformations. RMSD values below 2.0 Å with respect to the corresponding atomic structures were considered favorable. At the beginning of the simulation, there was a sharp increase in the RMSD values for all compounds as the interactions attempted to reach equilibration. To reach a stable condition (equilibration), andrographolide required less than 1 ns. We extended the time but could not find any interesting features. The stability of the ligand-protein interactions was examined using RMSF values. In contrast to the RMSD, the RMSF was calculated for each residue to determine the extent of fluctuation of each residue during the simulation. In general, the RMSF provides the fluctuations of individual atoms during the simulation. The RMSF values for all ligand-protein simulations are shown in Fig. 7B, with the green line indicating the interacting residues. Based on the RMSF evaluation, some protein residues of andrographolide showed MD simulation fluctuations greater than 2.5 Å. Further observation showed that the residue fluctuated more than 2,5 Å, which was not the interacting residue. All interacting residues, indicated by a green line, generated RMSF values below 2.5 Å; thus, it is considered acceptable, indicating that the ligand–protein interactions are stable. Protein-ligand contact fraction: In this subsection, protein-ligand contact was investigated to further validate docking scores and MM-GBSA values. The protein-ligand contacts during the simulation were recorded and processed as a simulation interaction diagram and timeline representation. These results provide information on the types, fractions, and residues involved. The interaction fraction indicates the fraction of the simulation time during which specific interactions occurred. For instance, an interaction fraction of 0,6 means that a residue interacted with the ligand for 60% of the simulation time, or in this study, 100ns molecular dynamics equal to 6ns. A value of more than 1,0 is possible for a single residue, as it may contact multiple groups in a ligand **(**Fig. 7C). Andrographolide showed the presence of 29 contact residues, such as Gln 146, Asn 145, Tyr 142, Asp 141, Gln 8, Asn 8, Asn 6, Pro 4, Gln 3, Ala 2, Met 1, Ser 0, Ser 1, Tyr 29, Ala 32, Ser 33, Met 34, Gln 35, Pro 36, Ile 37, Ser 297, Gln 298, Tyr 300, Gly 301, Arg 304, Asp 305, Gln 321, Pro 322, And Glu 325, at 0 hass the highest fraction produced from hydrogen bonds and water bridges.

### Andrographolide inhibits colorectal cancer cell growth *in-vitro*

**Fig. 8:**
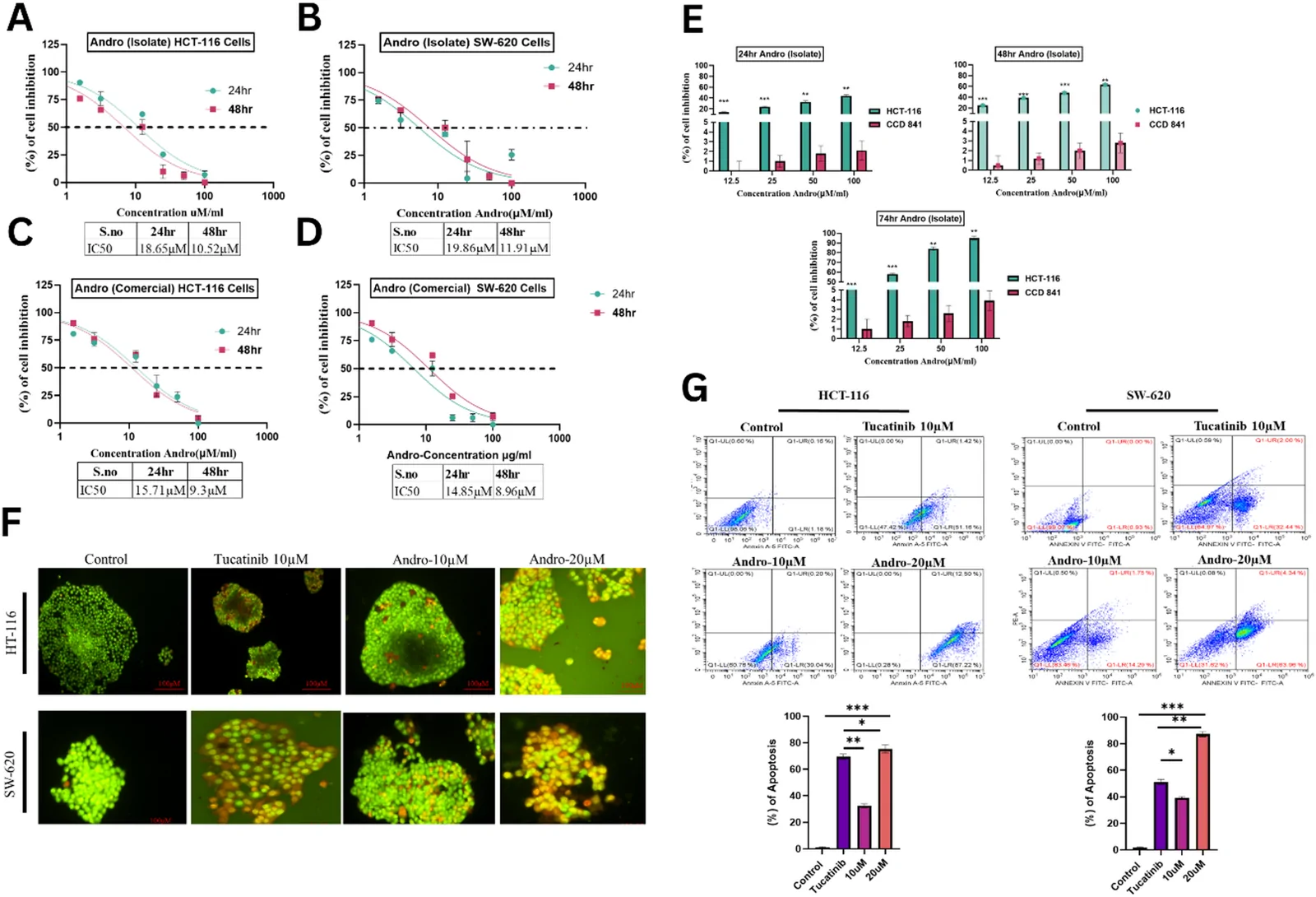
Comparative assessments of commercially available Andrographolide with that of the Andrographolide purified from *S. cusia* (**A** and **B**). Andro isolate was treated with HCT-116 and SW-620 for 48 h. (**C** and **D**) Andro commercial were treated with HCT-116 and SW-620 for 48 h. (**E**) The ethanol extract was more selective for the colorectal carcinoma cell line HT-116 than for the normal lung epithelial cell line CCD 841. To determine the therapeutic index of the ethanol extract, normal colon epithelial cell line CCD 841 and colorectal carcinoma cell line HT-116 were treated with increasing concentrations of the extract for 24 and 48 h. (**F**) Andro triggered apoptosis in HCT-116 and SW-620 cells (AO/EB staining). (**G**) Andro induced apoptosis in HCT-116 and SW620 cells. The data are expressed as SEM (*n* = 5) and statistical significance ***p > 0.0001, **p > 0.01, and *p > 0.05.

To further confirm that Fraction-6 contained andrographolide, its cytotoxic activity was compared with that of commercially available andrographolide (Sigma-Aldrich, St. Louis, MO, USA) in the HCT-116 and SW620 colorectal cancer cell lines. The growth-inhibitory effects of purified Fraction-6 and standard andrographolide were evaluated over 24 and 48 h. The results demonstrated comparable cytotoxic potency between the isolated compound and the commercial standard in both cell lines. Notably, treatment produced a dose- and time-dependent reduction in cell viability (Fig. 8A and B), supporting that Fraction-6 predominantly contains andrographolide and exhibits similar biological activity. The commercial Andro growth inhibition is shown in (Fig. 8C and D). In addition, the selectivity of Andro was tested against the CRC cell line HCT-116 and CCD 841 normal colon epithelial cells at 12.5,25, 50, and 100 M concentrations treated for 24, 48, and 72 h. The compound exhibited selectivity toward the HCT-116 cell line compared to CCD 841 cells (Fig. 8E). For example, after 48 h of treatment with 100 µg/ml of Andro, only1.4% growth inhibition was observed in CCD 841 cells compared to 61% in the case of HCT-116cells. Supporting the cytotoxicity of andrographolide in cancer cell lines, analysis of lactate dehydrogenase released into the cell culture media, an indicator of cell death, also showed that treatment with andrographolide induced death in colorectal cancer cells (Supplementary Figure S1).

### Assessment of andrographolide induces apoptosis

To investigate the apoptotic effects of andrographolide (Andro) on colorectal cancer cells, HCT-116 and SW-620 cell lines were subjected to AO/EB dual staining and observed using fluorescence microscopy. Viable cells emitted green fluorescence, whereas apoptotic cells with condensed chromatin exhibited orange-red fluorescence. Microscopic analysis revealed a significant, dose-dependent increase in apoptotic cells in the andro-treated groups compared with the untreated and vehicle control (1% DMSO) cells, indicating the effective induction of apoptosis (Fig. 8F). Furthermore, flow cytometric analysis using Annexin V-FITC and propidium iodide (PI) dual staining confirmed these findings, showing early and total apoptosis rates of 39.04% and 87.22%, respectively, in HCT-116 cells and 14.29% and 63.98%, respectively, in SW-620 cells. Notably, a marked increase in late-apoptotic cells was observed in HCT-116 cells, emphasizing the potent pro-apoptotic and anti-proliferative activities of andrographolide in a concentration-dependent manner, thereby supporting its potential as a natural therapeutic candidate against colorectal cancer.

## Discussion

Although the present study successfully integrated phytochemical profiling, network pharmacology, molecular docking, molecular dynamics simulations, and in-vitro validation to investigate the anti-colorectal cancer potential of *S. cusia* (Paul, 2021)^13, 17^ and highlighting novel therapeutic opportunities for CRC, certain methodological and biological limitations remain. Sequential extraction of SC leaves using solvents of varying polarities (n-hexane, chloroform, ethanol, and water) revealed distinct differences in the extraction yield and bioactivity. Among these, the ethanol extract exhibited the highest potency, with IC50 values of 54.37 µg/mL for HCT-116 and 77.52 µg/mL for SW-620 cells at 48 h, whereas the chloroform, hexane, and aqueous extracts exhibited weaker activity. Importantly, SCE demonstrated selective cytotoxicity toward CRC cells compared to normal colorectal epithelial CCD 841 cells, supporting its therapeutic potential. However, selectivity decreased with extended exposure, underscoring the importance of optimizing dosage and duration. These results align with those of earlier studies, indicating that ethanol efficiently extracts polar phytochemicals, such as flavonoids, terpenoids, and lactones, which are frequently implicated in anticancer activity (Paul, 2021). GC-MS profiling of the ethanol extract revealed the presence of fatty acids, esters, and heterocyclic compounds, consistent with previous phytochemical reports on *S. cusia*. Crucially, andrographolide, a labdane diterpenoid lactone previously sourced mainly from *Andrographis paniculata*, was successfully isolated and structurally confirmed in *S. cusia*for the first time^11, 13^. Characterization through UV–vis spectroscopy demonstrated maximum absorbance at 223 nm, while FTIR revealed a γ-lactone carbonyl stretch at 1727 cm⁻¹. The NMR spectra further validated the structure, which was consistent with the published reference data. This discovery expands the phytochemical repertoire of SC and introduces it as a novel alternative reservoir for andrographolide, offering a potential solution to the supply limitations associated with traditional sources. Purified andrographolide significantly inhibited CRC cell proliferation in a dose- and time-dependent manner, with efficacy comparable to that of commercial standards. It also displayed selectivity toward cancer cells, producing minimal inhibition of normal CCD 841 colon epithelial cells, even at higher concentrations of the compound. Supporting assays confirmed this effect: lactate dehydrogenase release demonstrated cytotoxicity, acridine orange/ethidium bromide staining revealed a high proportion of apoptotic cells, and colony formation assays showed markedly reduced clonogenic survival. Flow cytometry further confirmed robust apoptosis induction, with up to 87% of apoptotic cells observed in HCT-116 cells following treatment (Paul, 2021^13^;. Collectively, these findings establish andrographolide as a potent and selective anticancer agent against CRC.

To gain mechanistic insights, network pharmacology was employed to explore the multi-target potential of *S. cusia*phytoconstituents^23^. Thirteen compounds were identified from the GC-MS data and curated databases, and their drug-likeness was assessed using Lipinski’s rule of five. The integration of compound-associated targets with CRC-related genes from GeneCards revealed seven overlapping targets: JAK1, CDK2, PTGS1, WEE1, CHEK1, CSF1R, and KDR. These proteins regulate key hallmarks of CRC, including cell cycle progression, DNA repair, apoptosis, and angiogenesis. The protein–protein interaction network identified CDK2 as a central hub, closely linked to CHEK1, WEE1, and JAK1, reflecting the pivotal role of checkpoint kinases in sustaining the uncontrolled proliferation of cancer cells. KEGG enrichment highlighted the PI3K–Akt, JAK–STAT, and cell cycle regulation pathways, whereas gene ontology analysis underscored their roles in cell death regulation, apoptotic signalling, and tyrosine phosphorylation. Collectively, these findings support the polypharmacological properties of *S. cusia*extracts^15, 17^. However, the network pharmacology analysis relied primarily on publicly available databases and predictive computational algorithms, which may contain incomplete annotations, prediction bias, or false-positive interactions.

Molecular docking studies demonstrated that andrographolide and related *S. cusia*phytoconstituents bind strongly to CHEK1, a serine/threonine kinase critical for the DNA damage response^5, 24^. The tri-trimethylsilyl derivative of andrographolide exhibited the strongest affinity (–6.40 kcal/mol), forming hydrogen bonds with TYR A:31 and ASN A:28 and van der Waals interactions with GLU A:33, TYR A:71, HIS A:73, and GLY A:72 residues. Ethyl iso-allocholate and 2,4-di-tert-butylphenol also exhibited strong affinities, whereas weaker binders, such as behenic alcohol, lacked significant hydrogen bonding. ADMET predictions suggested favorable drug-like properties, including oral bioavailability, intestinal absorption, and blood–brain barrier permeability, with minimal predicted toxicity. Four compounds, including andrographolide, satisfied Lipinski’s criteria^25^. MD simulations confirmed these docking predictions, with RMSD values consistently below 2 Å, indicating stable ligand–protein complex formation. RMSF analysis confirmed minimal fluctuations in interacting residues, whereas contact fraction analysis showed persistent hydrogen bonding and water-bridged contacts throughout the simulation^8^. These findings strongly support CHEK1 inhibition as a plausible anticancer strategy. Nevertheless, molecular docking and MD simulations provide predictive insights under simulated conditions and may not fully reflect the complexity of biological systems in-vivo.

Mechanistically, andrographolide appears to inhibit CRC proliferation through dual mechanisms involving direct CHEK1 inhibition and activation of apoptotic pathways. CHEK1 inhibition disrupts DNA damage checkpoints, preventing CRC cells from arresting at the G2/M phase and repairing DNA damage, thereby sensitizing them to apoptosis Paul, 2021^11^. Flow cytometry confirmed mitochondrial-mediated apoptosis, and reduced clonogenic survival suggested durable cytostatic effects. Network pharmacology further highlighted interactions with JAK1 and CDK2, implying pleiotropic modulation of multiple oncogenic pathways. This aligns with the multitarget behaviour of natural products, which can simultaneously modulate several signalling nodes and potentially offer therapeutic advantages over single-target drugs^17^.

These findings extend the existing literature on the anticancer activity of andrographolide. Previous studies demonstrated efficacy against breast, pancreatic, and hepatocellular carcinomas primarily through NF-κB inhibition, PI3K–Akt suppression, and reactive oxygen species induction. Recent CRC studies (Zhao et al., 2022; Nguyen et al., 2023; Sun et al., 2024) reported inhibition of proliferation and enhanced chemosensitivity in CRC cells. Our study provides novel evidence identifying CHEK1 as a direct molecular target of *S. cusia*-derived andrographolide in CRC, supported by docking and MD simulations^5, 25^. Furthermore, the discovery of andrographolide in*S. cusia* expands its natural sources, ensuring broader accessibility and supporting future therapeutic development. Despite these promising results, several limitations of this study must be acknowledged. Furthermore, only selected phytoconstituents and signaling pathways were experimentally validated, whereas the synergistic or antagonistic interactions among multiple bioactive compounds present in the extract were not comprehensively investigated.] While docking and MD simulations strongly suggested stable CHEK1 inhibition, definitive confirmation requires biochemical assays such as kinase activity measurements or western blotting of checkpoint signalling proteins. Additionally, in-vitro assays provide only initial evidence; in-vivo studies in xenograft or orthotopic CRC models are needed to evaluate pharmacokinetics, systemic toxicity, and therapeutic efficacy (Wu et al., 2025). Moreover, despite the selective anticancer activity of andrographolide, its poor aqueous solubility and potential metabolic instability may hinder clinical applicability and require further optimization through nano formulations, structural modifications, or prodrug-based strategies. Therefore, future investigations involving biochemical validation assays, mechanistic studies, bioassay-guided fractionation, animal models, and multi-omics approaches are necessary to comprehensively validate the therapeutic potential and molecular mechanisms of *S. cusia*-derived phytoconstituents against colorectal cancer. This study establishes *S. cusia* as a novel source of andrographolide and demonstrates its selective anticancer effects against colorectal cancer. Through an integrative approach combining phytochemical characterization, network pharmacology, molecular docking, MD simulations, and in-vitro assays, strong evidence was obtained supporting CHEK1 inhibition as a key mechanism underlying the activity of andrographolide, with additional interactions involving JAK1 and CDK2. These findings underscore the translational potential of S. casia derived andrographolide as a multi-targeted therapeutic candidate for CRC and highlight the importance of further in-vivo validation and formulation optimization to advance its clinical application.

## Materials and methods

The cell lines HCT-116 and SW-620 were procured from NCCS, Pune, Maharashtra, India. The CCD 841 cell line was obtained from the American Type Culture Collection (CRL-1790). Cell culture reagents and disposables were obtained from Life Technologies (Carlsbad, CA, USA). BSA was obtained from Hi-Media (Mumbai, Maharashtra, India), analytical grade n-hexane, Chloroform, Ethyl acetate, Ethanol, Silica gel 60–120, and ALUGRAM-Xtra aluminum plate SIL G/UV 254 were obtained from Loba Chemie (Mumbai, Maharashtra, India), and a lactate dehydrogenase activity assay kit (cat: K730–500) was purchased from Bio Vision (Milpitas, CA, United States). Acridine orange, ethidium bromide, and sodium butyrate were obtained from Sisco Research Laboratory Pvt. Ltd. (Mumbai, Maharashtra, India).

### Software and hardware configuration

Web-based data mining and network construction were conducted using Cytoscape and STRING for protein–protein interaction (PPI) network analysis. A modeling visualizer was used to facilitate data analysis and visualization. All computational analyses were performed on a system running Windows 10, equipped with an Intel^®^ Core™ i3-8130U CPU @ 2.20 GHz, 16 GB of RAM, and a 64-bit operating system with an x64-based processor.

### Extraction and isolation

#### Preparation and extraction of *S. cusia* leaves

Fresh, healthy leaves of *S. cusia*were collected from naturally growing roadside populations in Assam, northeastern India. Plant material was collected from non-protected areas in accordance with local regulations. The species was taxonomically identified and authenticated by Dr. C.M. Murugan, Botanical Survey of India (BSI), (Southern Regional Center, Coimbatore, India). A voucher specimen was deposited in the Herbarium of the Botanical Survey of India, Southern Regional Center, Coimbatore, India, under deposition number (BSI/SRC/5/23/2024/Tech/1986). Following taxonomic confirmation, the leaves were subjected to a standardized preparation process. Initially, they were thoroughly washed with tap water to eliminate dust, soil particles, and other contaminants. This was followed by gentle rinsing with distilled water to ensure the complete removal of residual impurities. The leaves were then air-dried in the shade until fully dehydrated to preserve their bioactive compounds. Once dried, the leaves were ground into a fine powder using a sterile electric blender, making them suitable for subsequent experiments^26^.

####  Polarity-based sequential extraction of leaf powder

The dried leaf powder of *S. cusia* was subjected to successive extraction using a Soxhlet apparatus. Each extraction employed 500 mL of different solvents in sequence: 70% aqueous ethanol, hexane, water (aqueous), and chloroform, to ensure that a broad range of phytochemicals were isolated. After extraction, the solvents from each fraction were removed under reduced pressure using a rotary evaporator for 24 h to obtain concentrated extracts. The resulting residues were stored at −80°C for 48 h to preserve their bioactive components, followed by lyophilization to yield a stable, dry extract suitable for further analyses^27^. Stock solutions of each lyophilized sample were prepared by reconstituting 10 mg of the material in 1 ml of 100% dimethyl sulfoxide (DMSO). The stock solutions were stored at 4 °C until use.

#### Screening of extracts for anti-cancer activity

The anticancer activity of the *S. cusia*crude samples was assessed using the MTT colorimetric assay in human colorectal carcinoma cell lines HCT-116 and SW-620. Cells (1.0 × 10⁵ cells/mL) were seeded into 96-well plates in L-15 medium supplemented with 10% FBS (Thermo Fisher Scientific, Cat. 26140079) and 1% penicillin-streptomycin (Thermo Fisher Scientific) and 5% GlutaMAX (Thermo Fisher Scientific, Cat. No. 35050061), and incubated at 37 °C with 5% CO₂ and 95% relative humidity until reaching 60 to 70% confluency 48 h. Cells were then treated with the extracted samples (1–1000 µg/mL), dissolved in DMSO (Thermo Fisher Scientific, Cat. No. D12345) and diluted in DMEM with 10% FBS, ensuring a final DMSO concentration of 0.1% for 24, 48, and 72 h post-treatment. Following treatment, 20µL of MTT solution (5 mg/mL in PBS) was added to each well and incubated for 3–4 h at 37 °C to allow viable cells to reduce MTT into purple formazan crystals. The medium was then removed, and the crystals were solubilized with 150µL of DMSO, followed by gentle shaking for 10–15 min. Absorbance was measured at 570 nm using a microplate reader (Multiskan Sky, Thermo Fisher Scientific)^28^^,29^.

#### GC-MS analysis

GC-MS analysis was performed on the samples using Shimadzu’s GC-MS QP2010 series operating in electron impact ionization mode, fitted with a BPX5 GC column (30 m length, 0.25 μm film thickness, 0.25 mm internal diameter). High-purity helium gas (99.999%) served as the carrier gas at a constant flow rate of 1 mL/min, with an injection volume of 1µL at a split ratio of 10:1. The ion source temperature was set to 200 °C, and the injector temperature was set to 250 °C. The oven temperature program commenced at 60 °C, held for 1 min, then increased to 300 °C at 15 °C/min and maintained for 30 min. The solvent delay was configured from 0 to 45 min, and the mass spectrometer operated in positive electron ionization mode at 70 eV, scanning fragments in the m/z range of 35–500 Da. The relative percentages of the components were determined based on the peak area/total peak area ratios. Data processing was performed using LabSolutions software, with compound identification referenced against the NIST Main 4.0 library (National Institute of Standards and Technology)^30^.

#### Andrographolide isolation

Five grams of *S. cusia*extract was dissolved in 10 mL of methanol and applied to analytical thin-layer chromatography (TLC) plates pre-coated with silica gel (Merck, Mumbai, India). Several solvent systems were evaluated to optimize separation, including chloroform (100%), chloroform: ethanol (9:1), ethanol: ethyl acetate (1:6), and chloroform: methanol: ethyl acetate (1:6:3). Among these, pure chloroform produced the highest number of resolved bands, indicating efficient separation of the phytochemical constituents. The methanol used to dissolve the crude extract was evaporated, and the residue was washed with chloroform to remove nonpolar and pigmented compounds. Preliminary phytochemical screening using the Salkowski test revealed a brownish-red ring, confirming the presence of terpenoids, and a distinct TLC band corresponding to andrographolide. For further purification, the crude extract was adsorbed onto 2 g of silica gel (mesh size 60–120, HI Media, Mumbai, India) and subjected to column chromatography (CC). The column parameters were an internal diameter of 4.5 cm, stationary phase length of 20 cm, and sample load height of 1.5 cm. The mobile phase consisted of chloroform: methanol: ethyl acetate in a ratio of 1:6:3. The first eluted fraction exhibited a TLC profile that matched that of the andrographolide standard (Sigma-Aldrich, USA). Subsequently, preparative TLC was performed for further purification. Five grams of silica gel (mesh size 60–120, HI Media, Mumbai, India) was suspended in 10 mL of distilled water and uniformly coated onto a glass TLC plate. The silica layer was activated by drying in a hot air oven at 80 °C overnight. The andrographolide-containing fraction was applied as a streak along one edge of the plate and developed using ethanol as the mobile phase. The resolved andrographolide band was carefully scraped off, dissolved in ethanol to separate it from silica, and the supernatant was dried at room temperature to obtain purified andrographolide crystals^31, 32^.

#### Spectral analysis

The maximum absorption in the UV-visible region of the electromagnetic spectrum was determined using a 1 mg/mL solution of isolated andrographolide prepared in methanol, which was scanned over the wavelength range of 190–800 nm using a UV-visible spectrophotometer (JASCO V-750, Japan). The melting point of the compound was assessed using a standard melting-point apparatus. Functional groups were identified via Fourier Transform Infrared (FTIR) spectroscopy employing IR transmission spectra, where a potassium bromide (KBr) disc incorporating 5% of the sample was prepared and scanned across the wavenumber range of 400–4000 cm⁻¹ using a (Thermo Fisher Scientific Nicolet 6700 spectrometer, USA). For structural confirmation, proton nuclear magnetic resonance (¹H NMR) at 399.8 MHz and carbon-13 nuclear magnetic resonance (¹³C NMR) at 100.53 MHz were conducted in deuterated dimethyl sulfoxide (DMSO-d₆)^33, 34^.

### Network pharmacology

The network pharmacology workflow previously established guidelines for herbal medicine-based network pharmacology research^22^. Colorectal cancer-associated target genes were retrieved from the GeneCards database using the keyword “colorectal cancer.” To ensure biological relevance and minimize weak disease associations, only genes with GeneCards Inferred Functionality Scores (GIFtS) > 60 and relevance scores above the median value were retained, and duplicate entries were removed before downstream analysis. Target genes corresponding to the selected*S.cusia* phytoconstituents were predicted using the SuperPRED database with Homo sapiens selected as the target species, and only targets with probability scores (≥ 0.1) were retained to improve prediction reliability. The identified targets were subsequently mapped to STRING identifiers using STRING v12.0 to ensure standardized protein annotation and compatibility with downstream analyses.

Common targets between phytoconstituent-associated genes and colorectal cancer-related genes were identified using Venny v2.1 by overlapping the two datasets and extracting intersecting genes from the generated Venn diagram. Protein–protein interaction (PPI) analysis was performed using STRING v12.0 with Homo sapiens selected as the target organism and a high-confidence interaction score threshold of (> 0.9) to minimize false-positive interactions and improve network reliability^35^^,19^. The resulting PPI network consisted of nodes representing proteins and edges representing protein interactions. Network visualization and topological analyses were performed using Cytoscape (version 3.10.1) integrated with the STRING plugin (version 2.1.1). Hub genes were identified using the CytoHubba plugin based on the Maximal Clique Centrality (MCC) algorithm. GO and KEGG enrichment analyses were performed using ShinyGO v0.8.0 by employing the intersecting genes identified from the Venny analysis as the input dataset, while the complete*Homo sapiens* genome available in ShinyGO was used as the background reference set. Statistical significance was determined using a false discovery rate (FDR) cutoff of < 0.05, and enrichment P-values were adjusted using the Benjamini–Hochberg multiple-testing correction method. Pathways were prioritized and selected for reporting based on enrichment significance, gene count, and known biological relevance to colorectal cancer progression and associated signaling pathways.

### Molecular docking analysis

The crystal structure coordinates of CHEK1 (PDB ID: 1NVQ) were retrieved from the RCSB Protein Data Bank (PDB). Protein preparation was performed using Schrödinger’s Protein Preparation Wizard in Maestro, including correction of structural defects, hydrogen-bond optimization, and restrained minimization with the OPLS force field using a convergence criterion of 0.3 Å RMSD. Molecular docking was carried out using the Glide protocol in Maestro software. The three-dimensional structures of the test compounds were prepared and energy-minimized using LigPrep with the OPLS force field, applying 5000 iterations and a minimum RMSD gradient of 0.10. Receptor grids were generated based on the co-crystallized ligand binding site. Docking simulations were conducted using Glide Ligand Docking in standard precision (SP) mode with a rigid receptor and flexible ligand sampling approach^5, 10^. To validate the docking workflow and enable comparative ligand–target analysis, the known CHEK1 inhibitor Prexasertib was included as a positive control and docked against CHEK1 using the same protein preparation protocol, receptor grid dimensions, binding site coordinates, ligand preparation settings, and Glide SP docking parameters applied for all phytoconstituents. Docking validation was further performed by redocking the co-crystallized ligand into the active site of CHEK1. The RMSD between the experimental and predicted poses was 0.78 Å, which was below the accepted threshold of 2.0 Å, confirming the reliability of the docking parameters. In addition, superimposition of the prepared and raw CHEK1 structures yielded an RMSD below 2 Å, while docked ligands showed RMSD values below 1.5 Å, further validating the robustness and accuracy of the docking workflow for screening*S. cusia*-derived phytoconstituents.

### In-silico drug-likeness and ADME-tox predictions

The drug-likeness parameters of the phytoconstituents were evaluated using the Target Net web portal (targetnet.scbdd.com). The nine most active constituents of *S. cusia*, selected based on their superior docking scores, were assessed for compliance with Lipinski’s Rule of Five (RO5). Potential novel molecules were identified according to established drug-like criteria, including molecular refractivity (MR) within 40–130, molecular weight (MW) ≤ 500 g/mol, hydrogen bond donors (HBD) ≤ 5, hydrogen bond acceptors (HBA) ≤ 10, and lipophilicity (log P) ≤ 5^25, 36^. In-silico absorption, distribution, metabolism, excretion, and toxicity (ADME-Tox) profiles were generated using the pre-ADMET web service (preadmet.webservice.bmdrc.org), providing insights into the pharmacokinetic properties of the candidate molecules. This analysis facilitated the prioritization of compounds exhibiting favorable drug-like attributes for subsequent experimental validation. Specifically, ligands with elevated docking scores were scrutinized for key pharmacokinetic parameters, including blood-brain barrier (BBB) permeability, plasma protein binding affinity, aqueous solubility, and carcinogenic potential. These evaluations enabled the identification of active ingredients from S. glauca, alongside the reference compound tacrine, that align with drug-likeness and ADME-Tox criteria, thereby informing the rational design of novel therapeutics with optimized pharmacological profiles.

### Molecular dynamics simulation

Before running the molecular dynamics simulation, the system was prepared using the System Builder tool in Schrödinger Desmond to define the appropriate parameters. The protein–ligand complexes were placed in an orthorhombic simulation box with buffer dimensions of 10 Å in each direction to ensure adequate solvation and minimize the box volume. The system was solvated with the Simple Point Charge (SPC) water model, and ion neutralization was achieved by adding counterions (Na⁺). Physiological conditions were simulated by introducing salt at a concentration of 0.15 M NaCl. Energy minimization was performed, after which MD simulations were carried out under the NPT ensemble, maintaining a constant temperature of 310 K (using the Nose–Hoover thermostat) and a constant pressure of 1.013 bar (using the Martyna–Tobias–Klein Barostat). Each simulation was run for 10 ns, and trajectory snapshots were saved at 0.1 ns intervals. The generated trajectories were analyzed for root mean square deviation (RMSD), root mean square fluctuation (RMSF), and protein–ligand contact stability to evaluate conformational dynamics and interaction stability^8, 37^. The outputs were further visualized to interpret the structural stability and binding interactions during the simulation period.

### In-vitro sssessments

#### MTT sssay

The antiproliferative activity of andrographolide against colorectal carcinoma cell lines HCT-116 and SW-620 and the non-cancerous human colon epithelial cell line CCD-841 was evaluated using the above protocols. The cells were seeded into 96-well plates in L-15 medium supplemented with 10% FBS, 1% penicillin/streptomycin, and 5% GlutaMAX (Thermo Fisher Scientific, Cat. No. 35050061), and incubated at 37 °C with 5% CO₂ and 95% relative humidity until reaching 60 to 70% confluency after 48 h. Cells were then treated with andrographolide (1–100 µg/mL), dissolved in DMSO, and diluted in DMEM with 10% FBS, ensuring a final DMSO concentration of 0.1% for 24 and 48 h. Following treatment, 20µL of MTT solution (5 mg/mL in PBS) was added to each well and incubated for 3–4 h at 37 °C to allow viable cells to reduce MTT into purple formazan crystals. The medium was then removed, and the crystals were solubilized with 150µL of DMSO, followed by gentle shaking for 10–15 min. The percentage of cell viability calculations using GraphPad Prism (Version 10.2.0).

#### Morphological assay

HCT-116 and SW-620 cell lines (1.0 × 10⁵ cells/mL) were treated with varying concentrations of PBS, Tucatinib (TU), and andrographolide (Andro) in a 96-well plate and incubated for 24 h at 37 °C in a humidified atmosphere containing 5% CO₂. The samples in the 96-well plates were divided into four groups, with 24 wells per group corresponding to different reagent concentrations. After 24 h of culture, 20µL of trypsin was added to each well. After cell detachment, 25µL suspensions were transferred to glass slides. A 1µL dual fluorescent staining solution containing 100 µg/mL acridine orange and 100 µg/mL ethidium bromide (AO/EB, Sigma, St. Louis, MO) was added to each suspension, followed by a coverslip. Apoptotic cell morphology was examined by counting 500 cells within 20 min using a fluorescence microscope (Olympus, Tokyo, Japan). The dual acridine orange/ethidium bromide staining procedure was performed at least thrice^7, 38^.

#### Apoptosis assay

HCT-116 and SW-620 cells (1.5 × 10⁵ cells/well) were cultured in 6-well plates at 37 ± 0.5 °C with 5% CO₂ for 24 h in the presence of IC₅₀ concentrations of Andro, TU, or control PBS. After incubation, the treated cells were trypsinized and washed three times with ice-cold phosphate-buffered saline. The cells were then resuspended in 100 µL of DNA-binding buffer for 5 min, followed by staining with 5 µL of Annexin V and 10 µL of PI for 10 min at ambient temperature (25 °C). Flow cytometry was performed using a CytoFLEX flow cytometer (Beckman Coulter, Inc., USA), and the data were analyzed accordingly^7, 39^.

### Statistical analysis

Statistical analyses were performed to evaluate the differences between the various drug treatments. One-way analysis of variance (ANOVA) followed by Student’s t-test was conducted using Prism 10.0.1 software (GraphPad, San Diego, CA, USA). Differences were considered statistically significant at *P* < 0.05.

## Supplementary Information

Below is the link to the electronic supplementary material.


Supplementary Material 1



Supplementary Material 2


## Data Availability

The authors confirm that the data supporting the findings of this study are available in the article and its Supplementary Materials.
