# Supplementary material for: Uncovering the potential anti-cancer compounds from Strobilanthes cusia against colorectal cancer through network pharmacology and molecular docking
Source: Sci Rep. 2026 Jun 15;16:27353. doi: 10.1038/s41598-026-56319-6 (PMC13529646; doi:10.1038/s41598-026-56319-6)
Supplement: Supplementary file 1 [file 41598_2026_56319_MOESM1_ESM.docx]

**Supplementary Figure S1:** Molecular Docking analysis of the 2D projection of *S. cusia* phytoconstituent interactions with CHEK1 Protein.

**Supplementary Figure S2:** Andrographolide and positive control triggers the release of lactate dehydrogenase, an indication of cell death into the medium. the data were expressed as SEM (n=5), ***p>0.0001, **p>0.01, and *p>0.05.
