# Supplementary material for: Uncovering the potential anti-cancer compounds from Strobilanthes cusia against colorectal cancer through network pharmacology and molecular docking
Source: Sci Rep. 2026 Jun 15;16:27353. doi: 10.1038/s41598-026-56319-6 (PMC13529646; doi:10.1038/s41598-026-56319-6)
Supplement: Supplementary file 2 [file 41598_2026_56319_MOESM2_ESM.doc]

| **RT (min)** | **Compound Name** | **CAS No.** | **Formula** |
| --- | --- | --- | --- |
| 9.3101 | Hexadecane, 1,1-bis(dodecyloxy)- | 56554-64-4 | C40H82O2 |
| 12.1102 | Behenic alcohol | 661-19-8 | C22H46O |
| 12.2124 | Octadecane, 3-ethyl-5-(2-ethylbutyl)- | 55282-12-7 | C26H54 |
| 13.6605 | 2,4-Di-tert-butylphenol | 96-76-4 | C14H22O |
| 14.5918 | Hexadecane, 1,1-bis(dodecyloxy)- | 56554-64-4 | C40H82O2 |
| 14.6760 | Octadecane, 3-ethyl-5-(2-ethylbutyl)- | 55282-12-7 | C26H54 |
| 16.7189 | 6-Hydroxy-4,4,7a-trimethyl-5,6,7,7a-tetrahydrobenzofuran-2(4H)-one | 73410-02-3 | C11H16O3 |
| 16.8211 | Bacteriochlorophyll-c-stearl | 1000164-49-7 | C52H72MgN4O4 |
| 16.8932 | Octadecane, 3-ethyl-5-(2-ethylbutyl)- | 55282-12-7 | C26H54 |
| 17.3258 | Ethanol, 2-(9,12-octadecadienyloxy)-, (Z,Z)- | 17367-08-7 | C20H38O2 |
| 18.4555 | Benzothiazole, 2-(2-hydroxyethylthio)- | 4665-63-8 | C9H9NOS2 |
| 18.8461 | Oleic acid | 112-80-1 | C18H34O2 |
| 18.9001 | Ethyl iso-allocholate | 1000043-05-3 | C26H44O5 |
| 26.1412 | Octadecane, 3-ethyl-5-(2-ethylbutyl)- | 55282-12-7 | C26H54 |
| 27.9080 | dl-alpha-Tocopherol | 10191-41-0 | C29H50O2 |
| 29.6629 | Andrographolide, tri-(trimethylsilyl)- | 1000454-62-8 | C29H54O5Si3 |
| 30.5764 | 5H-Cyclopropa[3,4]benz[1,2-e]azulen-5-one derivative | 77573-36-5 | C28H37ClO11 |

**Supplementary Table 1:** Some Secondary Metabolites in *S.casia* (SC), Ethanolic Extract Were Identified Using GC-MS and the NIST 17 Spectral Database.

| S.no | **Functional Group** | **Isolated Andrographolide** | **Andrographolide**  **Reported** | **Reference** |
| --- | --- | --- | --- | --- |
| 1 | O–H stretching | 3398.01 | 3396.53 | Vilvest, J *et al.,* 2024 |
| 2 | C=O (lactone) | 1756.23 | 1752.81 | Vilvest, J *et al.,* 2024 |
| 3 | C=C stretching (alkene) | 1674.37 | 1675.44 | Vilvest, J *et al.,* 2024 |
| 4 | C–O stretching | 1258.01 | 1256.09 | Vilvest, J *et al.,* 2024 |
| 5 | CH2/CH3 bending | 1388.69 | 1387.07 | Vilvest, J *et al.,* 2024 |
| 6 | C–H stretching | 2881.51 | 2881.08 | Vilvest, J *et al.,* 2024 |

**Supplementary Table S2:** Comparison of FT-IR spectra of Andrographolide

| S.no | **Proton Position** | **Andrographolide isolate (CD₃OD)** | **Andrographolide Reported (CD₃OD)** | **Reference** |
| --- | --- | --- | --- | --- |
| 1 | H-1ax | 1.31 | 1.24 | Vilvest, J *et al.,* 2024 |
| 2 | H-1eq | 1.82 | 1.49 | Vilvest, J *et al.,* 2024 |
| 3 | H-2ax | 1.99 | 1.72 | Vilvest, J *et al.,* 2024 |
| 4 | H-2eq | 1.95 | 1.47 | Vilvest, J *et al.,* 2024 |
| 5 | H-3 | 3.44 | 3.15 | Vilvest, J *et al.,* 2024 |
| 6 | H-5 | 1.29 | 1.39 | Vilvest, J *et al.,* 2024 |
| 7 | H-6ax | 1.45 | 1.16 | Vilvest, J *et al.,* 2024 |
| 8 | H-6eq | 1.95 | 1.41 | Vilvest, J *et al.,* 2024 |
| 9 | H-7ax | 2.18 | 2.01 | Vilvest, J *et al.,* 2024 |
| 10 | H-7eq | 2.35 | 1.91 | Vilvest, J *et al.,* 2024 |
| 11 | H-9 | 1.99 | 2.18 | Vilvest, J *et al.,* 2024 |
| 12 | H-11a | 2.74 | 2.09 | Vilvest, J *et al.,* 2024 |
| 13 | H-11b | 2.63 | 1.84 | Vilvest, J *et al.,* 2024 |
| 14 | H-12 | Not detected | 6.86 | Vilvest, J *et al.,* 2024 |
| 15 | H-14 | 5.01 | 4.5 | Vilvest, J *et al.,* 2024 |
| 16 | H-15a | 4.48 | 4.47 | Vilvest, J *et al.,* 2024 |
| 17 | H-15b | 4.06 | 4.22 | Vilvest, J *et al.,* 2024 |
| 18 | Me-16 | 1.22 | 1.16 | Vilvest, J *et al.,* 2024 |
| 19 | H-17a | 5.05 | 4.88 | Vilvest, J *et al.,* 2024 |
| 20 | H-17b | 4.57 | 4.63 | Vilvest, J *et al.,* 2024 |
| 21 | Me-18 | 1.23 | 1.16 | Vilvest, J *et al.,* 2024 |
| 22 | H-19a | 3.98 | 3.58 | Vilvest, J *et al.,* 2024 |
| 23 | H-19b | 3.32 | 3.33 | Vilvest, J *et al.,* 2024 |

**Supplementary Table S3:** Comparison of ^1^H-NMR of Andrographolide

| **Andro Isolate** | | **Andrographolide** | |
| --- | --- | --- | --- |
| **S,no** | **13C Shift** | **13C Shift** | **Reference** |
| 1 | CH2 38.19 | CH2 37.4 | Vilvest, J *et al.,* 2024 |
| 2 | CH3 29.09 | CH2 29.1 | Vilvest, J *et al.,* 2024 |
| 3 | CH 80.97 | CH 80.0 | Vilvest, J *et al.,* 2024 |
| 4 | R-C4 43.7 | Q 43.9 | Vilvest, J *et al.,* 2024 |
| 5 | CH 56.38 | CH 55.4 | Vilvest, J *et al.,* 2024 |
| 6 | CH2 25.25 | CH2 24.5 | Vilvest, J *et al.,* 2024 |
| 7 | CH2 38.19 | CH2 38.3 | Vilvest, J *et al.,* 2024 |
| 8 | =C 149.37 | Q 148.04 | Vilvest, J *et al.,* 2024 |
| 9 | CH 56.38 | CH 56.5 | Vilvest, J *et al.,* 2024 |
| 10 | R-C4 39.02 | Q 39.3 | Vilvest, J *et al.,* 2024 |
| 11 | CH2 25.76 | CH2 25.1 | Vilvest, J *et al.,* 2024 |
| 12 | =C 148.81 | CH 147.0 | Vilvest, J *et al.,* 2024 |
| 13 | =C 129.85 | Q 130.3 | Vilvest, J *et al.,* 2024 |
| 14 | CH 66.70 | CH 66.1 | Vilvest, J *et al.,* 2024 |
| 15 | CH2 76.17 | CH2 75.4 | Vilvest, J *et al.,* 2024 |
| 16 | C=O 170.67 | Q 170.7 | Vilvest, J *et al.,* 2024 |
| 17 | CH2 109.26 | CH2 108.8 | Vilvest, J *et al.,* 2024 |
| 18 | CH3 23.42 | CH3 23.8 | Vilvest, J *et al.,* 2024 |
| 19 | CH2 65.02 | CH2 64.2 | Vilvest, J *et al.,* 2024 |
| 20 | CH3 15.58 | CH3 15.3 | Vilvest, J *et al.,* 2024 |

**Supplementary Table S4:** Comparison of ^13^C-NMR of Andrographolide

| **Disease** | **Gene Card** |
| --- | --- |
| Colorectal cancer | EGFR, ERBB2, FGFR3, FGFR2, ESR1, FGFR1, MTOR, IGF1R, CREBBP, AKT3, CHEK2, BRAF, MET, RET, ERBB3, ERBB4, MAP2K1, KDR, RAF1, PTPN11, MAP2K2, ATM, TP53, KRAS, SMAD4, CTNNB1, AKT1, KIT, PDGFRA, CDK4, ATR, CHEK1, MDM2, NOTCH1, STAT3, SMAD3, EZH2, MAPK1, MMP2, MMP9, DNMT1, TGFBR1, NFKB1, JAK2, CFTR, AKT2, PDGFRB, NTRK3, NTRK2, ABL1, PIK3CD, SLC2A1, JAK1, EPHB4, INSR, HDAC2, FLT3, IKBKB, HDAC6, BTK, ADAM10, SOD1, TYK2, CDK5, PSEN1, PTEN, PIK3CA, BMPR1A, TGFBR2, CCND1, HRAS, MYC, EP300, AR, PPARG, CASP8, PIK3R1, RAD51, AURKA, SMAD2, TGFB1, SMO, IDH1, CXCR4, FLT4, CDK6, PARP1, DNMT3A, STAT1, IDH2, EPHB2, AXL, HGF, HSP90AA1, PRKCA, MAPK14, PLK1, EPHA2, CTSD, HSPB1, PRKACA, STAT6, NOTCH2, PRKCD, CSF1R, AURKB, NT5E, HMOX1, HDAC4, LCK, MPO, JAK3, MYLK, NFKB2, ITGB3, BCR, PTPRC, PLCG2, UCHL1, AGTR1, RPS6KA3, PRKCG, GRIN2A, SNCA, GRIN2B, CAMK2A, PROC. |

**Supplementary Table S5.** Identification of genes related to colorectal cancer using Gene Card

| **Predicted pathway** | **Matching genes in our network** |
| --- | --- |
| Progesterone-mediated oocyte maturation | CDK2, SPDYE2, SPDYE2B, SPDYE6, SPDYE5, SPDYE16 |
| Oocyte meiosis | CDK2, SPDYE2, SPDYE2B, SPDYE6, SPDYE5, SPDYE16 |
| Cell cycle | CDK2, CHEK1, WEE1 |
| PI3K-Akt signaling pathway | KDR, CDK2, CSF1R, JAK1 |
| Viral carcinogenesis | CDK2, CHEK1, JAK1 |
| Human T-cell leukemia virus 1 infection | CDK2, CHEK1, JAK1 |
| EGFR tyrosine kinase inhibitor resistance | KDR, JAK1 |
| p53 signaling pathway | CDK2, CHEK1 |
| Progesterone-mediated oocyte maturation | CDK2, SPDYE2, SPDYE2B, SPDYE6, SPDYE5, SPDYE16 |
| TNF signaling pathway | SPDYE6, CDK2, CHEK1 |
| AMPK signaling pathway | SPDYE5, SPDYE16, CDK2, CHEK1, JAK1 |
| Thyroid hormone signaling pathway | SPDYE16, CHEK1, WEE1 |
| FoxO signaling pathway | SPDYE6, SPDYE5, SPDYE16 |
| Apoptosis | KDR, CDK2, CSF1R, JAK1 |
| Insulin signaling pathway | CDK2, CHEK1, JAK1 |

**Supplementary Table S6.** Identification of disease pathways matched to our common genes associated with the phytoconstituents and colorectal cancer through KEGG enrichment
